# SwiftMSeg: lightweight multi-scale local–global context modeling with transformer for medical image segmentation

**DOI:** 10.1038/s41598-026-56845-3

**Published:** 2026-06-07

**Authors:** Jahid Hasan Rony, Md Shakhawat Hossain, Fazlul Hasan Siddiqui

**Affiliations:** 1 Department of CSE, Dhaka University of Engineering and Technology, Gazipur, Bangladesh; 2 School of Informatics, Kochi University of Technology, Kami, 782-8502 Japan

**Keywords:** Computational biology and bioinformatics, Engineering, Mathematics and computing

## Abstract

Accurate medical image segmentation requires both fine boundary localization and robust contextual understanding, which is often difficult to achieve simultaneously, particularly in lightweight architectures. In this paper, we propose SwiftMSeg, a lightweight encoder–decoder framework that integrates a convolutional encoder, a transformer-based local–global–local module, and a hierarchical multi-scale decoder. The proposed framework addresses the boundary–context challenge by effectively combining progressive multi-scale refinement for fine boundary separation with global context modeling through long-range dependency aggregation. Extensive evaluations on publicly available colonoscopy, pathology, ultrasound, and magnetic resonance imaging datasets demonstrated the capability of SwiftMSeg to accurately segment diverse anatomical structures, ranging from tiny nuclei to polyps and large tumor regions. The model further demonstrated moderate domain-independent generalization on an external dataset, achieving Dice scores of 0.896 (colonoscopy), 0.860 (pathology), 0.850 (ultrasound), and 0.870 (MRI), consistently outperforming most baseline methods. In addition, it achieved improved boundary localization with lower Hausdorff distance (e.g., 16.43 in MRI and 33.89 in ultrasound) and reduced average symmetric surface distance, indicating more precise and stable segmentation. Statistical analysis further confirmed that the improvements of SwiftMSeg are significant (*p < 0.001*) with large effect sizes across modalities, validated by both paired *t*-tests and Wilcoxon tests. Despite its strong performance, SwiftMSeg remains highly efficient, requiring only 4.48M parameters and 0.940 giga floating-point operations per second (GFLOPs), reducing computational cost by approximately *∼*53*×* compared to the U-Net-based baselines (standard U-Net *∼*31M parameters and *∼*50 GFLOPs), while maintaining high segmentation accuracy. These results highlight the effectiveness of SwiftMSeg as a practical and scalable solution for real-world medical image segmentation across diverse modalities.

## Introduction

Medical image segmentation is a fundamental step in computer-aided diagnosis and quantitative image analysis, as it enables pixel-level segmentation of anatomical structures, organs, tissues, lesions, tumors, and other pathological targets to support quantitative assessment of disease burden, monitoring progression and treatment response and precise treatment planning. In recent years, deep learning has become the dominant approach for medical image segmentation, with encoder–decoder architectures emerging as a strong choice due to their strong performance through hierarchical feature extraction and progressive multi-scale reconstruction^1,2^. However, despite their success, several critical challenges remain in real-world medical deployment, including domain shifts across imaging devices and institutions, limited annotated data, class imbalance, and the need for accurate boundary delineation alongside robust global contextual understanding^3,4^. Furthermore, models trained for specific modalities or targets often exhibit poor generalization across different imaging conditions, such as histopathology, MRI, ultrasound, or endoscopic data^5,6^.

Convolutional neural networks (CNNs), which underpin most classical segmentation models, are inherently constrained by local receptive fields and therefore struggle to capture long-range dependencies. While deeper architectures partially mitigate this limitation through repeated pooling and feature aggregation, global contextual relationships are still modeled only implicitly. This limitation becomes particularly critical in medical imaging, where structures may exhibit large spatial variation, low contrast, or ambiguous boundaries. Notably, the nnU-Net^7^ model demonstrated that strong segmentation performance can often be achieved through systematic pipeline design rather than architectural complexity, highlighting the importance of efficiency-aware model development.

Transformers have recently emerged as a powerful alternative due to their explicit self-attention mechanism, which enables modeling of long-range dependencies and global semantic relationships^8^. Architectures such as Swin Transformer address the computational burden of global attention by introducing hierarchical and window-based strategies^9^. These advances have led to transformer-based segmentation frameworks, including UNETR and Swin-Unet, which integrate global context modeling into U-shaped architectures and demonstrate improved segmentation consistency^10,11^. Similarly, lightweight transformer designs such as SegFormer highlight the importance of balancing performance and computational efficiency through simplified decoders and multi-scale representations^12^. Nevertheless, transformer-based models often suffer from high computational cost, sensitivity to input resolution, and degradation of fine spatial details in dense prediction tasks^13^.

To address these limitations, recent research has focused on two key design directions: (i) enhancing local–global feature interaction and (ii) improving multi-scale decoding. Approaches such as ContextFormer^14^, Multi-level Feature Attention Networks^15^, and FeSeFormer^16^ introduce mechanisms to better integrate local spatial features with global contextual information. Meanwhile, advances in multi-scale decoding, including SegFormer^12^, Lawin Transformer^17^, and skip-attention-based^18^ designs, aim to recover fine spatial details and improve boundary localization. Despite these improvements, such architectures may increase model complexity, memory consumption or sensitivity to input variations.

Consequently, a key challenge remains in designing segmentation models that achieve a practical balance between accuracy, efficiency, and robustness. For real-world deployment in medical image analysis pipelines, models must not only deliver high accuracy but also maintain computational efficiency, as training is often constrained by limited GPU memory and clinical applications require low-latency inference on standard hardware. Lightweight CNN-based approaches often preserve local details but lack global coherence, whereas transformer-based methods provide strong contextual reasoning at the cost of increased computational overhead. Recent surveys emphasize the need for hybrid architectures that effectively combine local boundary preservation with global context modeling under realistic computational constraints^19,20^.

In this work, we propose a lightweight medical image segmentation framework that integrates multi-scale local–global feature modeling with a hierarchical decoding strategy. Rather than introducing a fundamentally new segmentation paradigm, this work focuses on improving the practical balance between contextual modeling, boundary refinement, and computational efficiency within a lightweight hybrid framework. Unlike lightweight hybrid architectures such as MobileViT and SegFormer, which primarily focus on efficient global representation learning, the proposed framework additionally emphasizes progressive hierarchical feature refinement for boundary-sensitive medical image segmentation. In particular, SwiftMSeg combines transformer-enhanced local–global aggregation with hierarchical multi-scale decoding to improve the balance between contextual understanding, spatial detail preservation, and computational efficiency. The proposed architecture is built around a local–global–local (LGL) module, which combines depthwise convolutions for preserving fine spatial details with transformer-based self-attention for capturing long-range dependencies. Fine boundary localization is achieved through a hierarchical multi-scale decoder that progressively restores spatial resolution and refines feature representations across scales. By jointly leveraging local feature extraction and global contextual modeling, the proposed approach bridges the gap between boundary-sensitive information and semantic coherence. This design enables computationally efficient segmentation while maintaining accurate and consistent predictions across diverse imaging modalities, making it suitable for real-world medical applications. The main contributions of this work are summarized as follows:

- A lightweight segmentation architecture that integrates local–global feature aggregation with hierarchical multi-scale decoding for improved contextual modeling and boundary refinement under low computational cost.
- A systematic ablation study demonstrating the complementary roles of encoder-level aggregation and decoder hierarchy in improving performance
- Robust and consistent segmentation across diverse modalities and anatomical structures, ranging from small nuclei to large tumors.
- A practical evaluation of medical image segmentation models by jointly assessing segmentation accuracy and deployment efficiency.

## Related works

Deep learning-based medical image segmentation has evolved from conventional CNN-based pipelines toward hybrid architectures that combine local spatial detail with global contextual modeling. Recent studies also emphasize efficiency-aware designs to address clinical deployment challenges such as domain shift, class imbalance, and limited computational resources^3,4^. Table 1 summarizes representative recent architectures and their design characteristics. In Table 1, the architectural characteristics are summarized qualitatively based on the primary design emphasis reported in the original studies. Here, “*∼*” (Partial) indicates that the corresponding property is indirectly addressed or only partially incorporated, rather than being a central or explicitly optimized component of the architecture.

**Table 1:** Summary of recent works and architectural focus (*HM* hierarchical multi-scale decoding, ✓: Yes, ✗: No, *∼*: Partially).

Model	Medical imaging	Transformer	Local–global	HM	Lightweight focus
SegFormer^12^	*∼*	✓	*∼*	*∼*	✓
UNet-InceptionV3^1,21^	✓	✗	*∼*	✓	✗
UNet-MobileNetV2^1,22^	✓	✗	*∼*	✓	✓
DeepLab-MobileNetV3^23,24^	*∼*	✗	*∼*	*∼*	✓
DeepLab-EfficientNetB0^23,25^	*∼*	✗	*∼*	*∼*	✓
UNet-EfficientNetB0^1,25^	✓	✗	*∼*	✓	✓
UNet-ResNet101^1,26^	✓	✗	*∼*	✓	✗
DeepLab-ResNet101^23,26^	*∼*	✗	*∼*	*∼*	✗
MA-Net^27^	✓	✗	*∼*	✓	*∼*
nnU-Net^7^	✓	✗	*∼*	✓	*∼*
UNETR^10^	✓	✓	*∼*	✓	✗
MedNeXt^28^	✓	✗	*∼*	✓	✓
VM-UNet^29^	✓	✓	*∼*	✓	✓
MA-TransformerV2^30^	✓	✓	✓	*∼*	✓
MK-UNet^31^	✓	✗	*∼*	✓	✓
SCM-UNet^32^	✓	✓	✓	*∼*	✓
**Proposed (SwiftMSeg)**	✓	✓	✓	✓	✓

### CNN-based encoder–decoder models

Convolutional architectures remain a cornerstone of medical image segmentation due to their strong spatial inductive bias and stable optimization. The U-Net^1^ introduced the skip-connection mechanism, which remains the gold standard for multi-scale feature fusion. Subsequent iterations, such as U-Net++^5^ and Attention U-Net^33^, focused on nested skip pathways and spatial attention to filter out non-target features. To leverage richer feature representations, U-Net has been widely combined with pretrained encoders such as ResNet^26^, InceptionV3^21^, EfficientNet^25^, and MobileNetV2^22^, offering varying trade-offs between segmentation accuracy and computational cost. Then, nnU-Net^7^ proposed another approach that focuses on automated pipeline configuration, including preprocessing and augmentation, without substantial architectural modifications. However, the inherent limitation of CNNs remains their narrow receptive field, which restricts their ability to model the long-range spatial dependencies critical for segmenting large or irregularly shaped pathological structures.

### Transformer-based segmentation models

To overcome the locality of CNNs, transformers introduce self-attention mechanisms that explicitly capture long-range dependencies and global semantic relationships^8^. In medical imaging, TransUNet^34^ first demonstrated the effectiveness of using a Transformer as an encoder backbone. UNETR^10^ and Swin-Unet^11^ further evolved this by employing hierarchical and window-based attention to reduce the quadratic computational complexity of standard self-attention. However, their direct application to high-resolution medical images is computationally expensive. To address this, hierarchical transformer architectures such as Swin Transformer employ window-based attention mechanisms to balance efficiency and contextual modeling^9^. Additionally, SegFormer presents a lightweight transformer-based approach that combines hierarchical representations with a simple decoder to achieve competitive performance with reduced complexity^12^. VM-UNet^29^ adopts Visual State Space (VSS) blocks based on the Mamba architecture to achieve linear-complexity global context modeling within a U-shaped encoder-decoder. Then MA-TransformerV2^30^ and SCM-UNet^32^ represent a more recent trend of explicitly coupling local convolutional features with global self-attention to address the boundary-localization weakness. Despite these gains, pure Transformer models often struggle with fine-grained boundary localization due to the loss of low-level spatial details during tokenization. This trade-off between global context and local boundary precision motivates the development of hybrid architectures^19^.

### Lightweight and local–global hybrid models

Recent research has increasingly focused on Local–Global–Local (LGL) information flow, capturing fine details, modeling global context and subsequently refining local boundaries. In parallel, another trend in medical image segmentation is the enhancement of the classical U-Net architecture through the integration of a powerful pre-trained backbone network. Instead of using a shallow encoder, modern variants adopt deep CNNs such as ResNet and EfficientNet to improve feature representation via transfer learning. For example, U-Net models with EfficientNet and ResNet backbones have demonstrated improved segmentation accuracy in tasks such as pneumothorax segmentation^35^, highlighting the importance of strong encoder representations. Similarly, attention-enhanced architectures such as DeepLabV3+ with attention mechanisms further emphasize the role of contextual modeling in improving segmentation performance^36^. Designing lightweight segmentation models that balance accuracy and efficiency is another major area of recent development. SegFormer^12^ and MobileViT^37^ paved the way for lightweight hybrid models that use depthwise convolutions alongside transformers to maintain efficiency. PlantCLR^38^ further highlights the effectiveness of lightweight transferable representation learning. DeepLabV3+ paired with efficient backbones such as EfficientNet-B0^23,25^ and MobileNetV3^23,24^ demonstrates how atrous spatial pyramid pooling can be made deployable under tight resource budgets, though the absence of skip connections limits fine boundary recovery. MA-Net^27^ approaches the local–global challenge from a purely convolutional standpoint using multi-scale channel attention and position-sensitive attention to fuse features across decoder levels without any transformer component. Similarly, recent weakly supervised approaches such as SGGAB-GAN incorporate superpixel-guided graph attention and adaptive feature refinement to improve boundary localization and contextual feature propagation under sparse scribble annotations^39^. Several studies have also explored alternatives to self-attention for modeling long-range dependencies. State space model (SSM)-based architectures, such as Vision Mamba and VM-UNet, offer scalable global modeling with reduced computational cost^29,40^. Similarly, MK-UNet^31^ highlights the continued relevance of lightweight CNN-based designs for resource-constrained environments, while SCM-UNet combines SSM-based global modeling with U-shaped decoding to improve segmentation performance^32^. Concurrently, advances in multi-scale decoding, including Lawin Transformer^17^ and skip-attention-based frameworks^18^, aim to enhance boundary localization and feature reconstruction. Beyond global contextual modeling, recent studies have further emphasized the importance of boundary fidelity and fine-detail preservation in medical image segmentation. For example, clDice introduced a topology-preserving loss function designed to improve structural connectivity in tubular segmentation tasks by explicitly preserving centerline continuity^41^. More recently, Frepa demonstrated that enhancing the representation of high-frequency components can substantially improve fine-grained anatomical detail preservation across diverse medical imaging tasks^42^. Similarly, HiPaS highlighted the clinical importance of accurate boundary-sensitive vessel segmentation through iterative multi-resolution refinement for pulmonary artery–vein delineation^43^. These studies further underscore the need for segmentation architectures that jointly optimize contextual understanding and precise boundary localization. Despite these advances, many backbone-enhanced and hybrid models improve feature representation at the cost of increased computational complexity and often do not explicitly address the boundary–context trade-off. This limitation motivates the need for architectures that jointly optimize local detail preservation, global context modeling, and computational efficiency within a unified framework.

### Summary and motivation

Table 1 summarizes the architectural properties of the representative models discussed above, highlighting that no existing method simultaneously satisfies all five criteria. The literature suggests a convergence toward hybrid architectures that integrate local detail preservation with global context modeling, often combined with multi-scale decoding strategies. However, many transformer-based models remain computationally demanding for real-time clinical use, lightweight models often sacrifice the high-resolution skip connections needed for precise boundary delineation and conventional decoders often fail to progressively refine features across multiple scales. Therefore, a critical research gap lies in designing architectures that achieve an effective balance between (i) fine-grained boundary preservation, (ii) long-range contextual modeling, and (iii) computational efficiency for practical deployment. The proposed framework addresses these challenges by integrating transformer-enhanced local–global feature aggregation with hierarchical multi-scale decoding to achieve a balanced trade-off between segmentation accuracy and computational efficiency.

## Materials and methodology

### Ethics statement

In this study, we have used four publicly available datasets. As all personal identifiers were removed and the images were fully de-identified. Therefore, the university research ethics committee waived the requirement for Institutional Review Board (IRB) approval. This study does not involve direct interaction with human participants or access to identifiable private information, thereby adhering to established ethical standards for human subjects research.

### Dataset

In this study, we trained and evaluated the models on four diverse medical imaging modalities: colonoscopy, pathology, ultrasound and MRI. These modalities were selected to provide a comprehensive and practical assessment of segmentation performance, as they represent distinct clinical applications with varying imaging characteristics, including differences in contrast, texture, noise, anatomical structures and target variability. Evaluating across these heterogeneous modalities ensures that the proposed model is robust and generalizable to a wide range of real-world medical imaging applications. For each modality, we used publicly available datasets for training and testing, conducting hold-out experiments, 4-fold cross-validation and ablation studies. In addition, external datasets were used to further evaluate model performance on unseen and heterogeneous data. The details of the datasets are described below and the data distribution for the experiments is presented in Table 2.

#### Colonoscopy images

For colonoscopy image segmentation, we used the Kvasir-SEG dataset ^44^, which contains 1,000 colonoscopy images with corresponding pixel-level masks for polyp segmentation. It was prepared at Vestre Viken Health Trust, Norway, with annotations created and verified by experienced gastroenterologists, ensuring high-quality ground truth. The dataset focuses on colorectal polyps, precursors to colorectal cancer, making it suitable for evaluating clinically relevant segmentation tasks. For external validation, we used the CVC-ClinicDB ^45^ to assess model performance on unseen clinical data. Both datasets include significant variability in polyp appearance and realistic challenges such as low contrast, reflections and occlusions, reflecting real clinical conditions.

#### Pathology images

For pathology image segmentation, we used H&E-stained histopathology images to segment cell nuclei, a fundamental and widely performed task in computational pathology. Nuclei segmentation is representative of target segmentation in pathology images, as it is essential for downstream analyses such as cell counting, cancer grading, and tumor microenvironment assessment, while being particularly challenging due to variations in nuclei size, shape, staining, tissue type and imaging conditions. We trained and evaluated our models on the NuInsSeg dataset ^46^, a widely used benchmark for nuclei segmentation that provides diverse and high-quality annotations verified by domain experts. For external validation, we used the PanNuke dataset ^47^, which contains multi-organ histopathology images with expert-annotated nuclei masks, enabling robust assessment of model generalization across different tissue types. These datasets include substantial variability in nuclei morphology and staining conditions, making them well-suited for evaluating the robustness and generalizability of pathology image segmentation models.

**Table 2:** Data distribution across four different medical image modalities.

**Split**	Colonoscopy	Pathology	Ultrasound	MRI
Train	600 (Kvasir-SEG)^44^	399 (NuInsSeg)^46^	1125 (BUS-BRA)^48^	2949 (BRISC)^49^
Validation	200 (Kvasir-SEG)^44^	133 (NuInsSeg)^46^	375 (BUS-BRA)^48^	984 (BRISC)^49^
Test	200 (Kvasir-SEG)^44^	133 (NuInsSeg)^46^	375 (BUS-BRA)^48^	860 (BRISC)^49^
External Test	80 (CVC-ClinicDB)^45^	80 (PanNuke)^47^	80 (UDIAT)^50^	80 (SciDB)^51^
**Total**	**1080**	**745**	**1955**	**4873**

#### Ultrasound images

For ultrasound image segmentation, we used breast ultrasound images to segment tumors, a clinically important task for computer-aided diagnosis aimed at early detection and characterization of breast abnormalities. Tumor segmentation in ultrasound is particularly challenging due to low contrast, speckle noise, heterogeneous lesion appearance and variability across imaging devices and patients, making it a representative task for evaluating target segmentation in ultrasound images. We trained and evaluated our models on the BUS-BRA dataset^48^, which contains biopsy-proven benign and malignant lesions, along with expert-annotated segmentation masks. For external validation, we used the UDIAT dataset^50^. Both datasets include substantial variability in tumor characteristics and imaging conditions, reflecting real clinical challenges.

#### MRI images

For MRI-based segmentation, we focused on brain tumor segmentation, a critical task for diagnosis, treatment planning and disease monitoring. Tumor segmentation in MRI is particularly challenging due to variability in tumor size, shape, location and intensity patterns across patients, making it a representative problem for evaluating target segmentation in MRI images. We trained and evaluated our models on the BRISC dataset ^49^, which consists of curated T1-weighted contrast-enhanced MRI scans with pixel-wise tumor annotations. The dataset includes multiple tumor types (glioma, meningioma, and pituitary tumors) and was carefully refined through sequence harmonization and expert verification by a radiologist and a physician to ensure label consistency and high-quality ground truth. For external validation, we used the SciDB dataset ^51^, which also contains T1-weighted contrast-enhanced MRI images from multiple patients with the same tumor types. Both datasets exhibit substantial variability in tumor characteristics and imaging conditions, providing a reliable benchmark for assessing the robustness and generalizability of MRI-based segmentation models.

### Proposed SwiftMSeg model

**Fig. 1:**
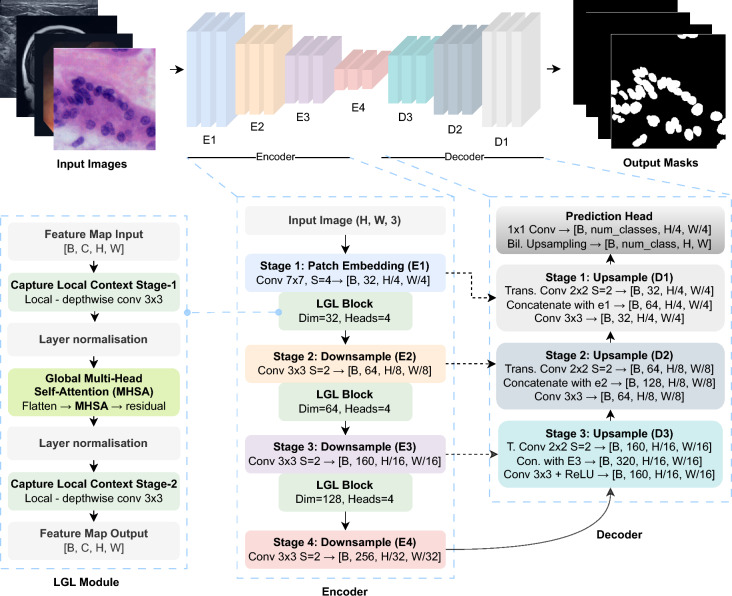
Architecture of the proposed (SwiftMSeg) model.

The proposed SwiftMSeg is a lightweight hybrid encoder-decoder architecture designed for accurate and efficient medical image segmentation. The architecture of SwiftMSeg is illustrated in Fig. 1. Given an input image $\textbf{X} \in \mathbb {R}^{H \times W \times 3}$, the model learns a mapping function $\mathcal {F}: \textbf{X} \rightarrow \textbf{Y}$, where $\textbf{Y} \in \mathbb {R}^{H \times W \times C}$ represents the pixel-wise segmentation logits for *C* classes. The architecture integrates convolutional operations for fine-grained spatial feature extraction with transformer-based self-attention for global context modeling. It follows a hierarchical encoder to learn multi-scale representations using overlapping patch embedding and Local–Global–Local (LGL) blocks, and a UNet-style decoder with skip connections to progressively restore spatial resolution and refine boundary details. This design effectively balances boundary localization and contextual understanding while maintaining low computational complexity.

#### Encoder with local–global–local (lGL) blocks

The encoder consists of four stages with progressive downsampling. The initial patch embedding is defined as:

1 $$\begin{aligned} \textbf{E}_1 = \phi _{7 \times 7, s=4}(\textbf{X}), \end{aligned}$$

where *ϕ* denotes a convolution operation with kernel size *7 × 7* and stride 4, producing feature maps at $\frac{H}{4} \times \frac{W}{4}$ resolution.

Subsequent stages apply strided convolutions:

2 $$\begin{aligned} \textbf{E}_{i} = \phi _{3 \times 3, s=2}(\textbf{E}_{i-1}), \quad i \in \{2,3,4\}, \end{aligned}$$

resulting in hierarchical features at $\frac{1}{8}$, $\frac{1}{16}$, and $\frac{1}{32}$ scales.

Each stage is enhanced using the proposed Local–Global–Local (LGL) block, which refines features through three steps:

1) Local Feature Extraction: A depthwise convolution captures spatially localized patterns:

3 $$\begin{aligned} \textbf{F}_l = \text {DWConv}(\textbf{E}), \end{aligned}$$

where $\text {DWConv}$ denotes depthwise convolution applied channel-wise.

2) Global Context Modeling: The feature map is reshaped into a sequence of tokens:

4 $$\begin{aligned} \textbf{Z} \in \mathbb {R}^{(HW) \times C}, \end{aligned}$$

and processed using multi-head self-attention:

5 $$\begin{aligned} \text {Attention}(\textbf{Q}, \textbf{K}, \textbf{V}) = \text {Softmax}\left( \frac{\textbf{Q}\textbf{K}^T}{\sqrt{d}}\right) \textbf{V}, \end{aligned}$$

where $\textbf{Q}=\textbf{K}=\textbf{V}=\textbf{Z}$. The output is combined with a residual connection:

6 $$\begin{aligned} \textbf{F}_g = \textbf{Z} + \text {MHSA}(\textbf{Z}). \end{aligned}$$

3) Local Refinement: The attended features are reshaped back and refined using another depthwise convolution:

7 $$\begin{aligned} \textbf{F}_{out} = \text {DWConv}(\text {Reshape}(\textbf{F}_g)). \end{aligned}$$

Thus, the LGL block can be summarized as:

8 $$\begin{aligned} \textbf{F}_{out} = \text {DWConv}(\text {MHSA}(\text {DWConv}(\textbf{E}))). \end{aligned}$$

#### Decoder with multi-scale feature fusion

The decoder follows a UNet-style design, progressively upsampling features:

9 $$\begin{aligned} \textbf{D}_{i} = \sigma \left( \text {Conv}\left( \text {Concat}(\text {Up}(\textbf{D}_{i+1}), \textbf{E}_{i}) \right) \right) , \end{aligned}$$

where $\text {Up}(\cdot )$ denotes transposed convolution, $\text {Concat}(\cdot )$ represents channel-wise concatenation, and $\sigma (\cdot )$ is a non-linear activation function.

The final segmentation output is obtained as:

10 $$\begin{aligned} \textbf{Y} = \text {Up}_{\text {bilinear}}(\text {Conv}_{1\times 1}(\textbf{D}_1)). \end{aligned}$$

#### Boundary localization and contextual understanding

Fine boundary localization is achieved through the hierarchical multi-scale decoder, which progressively restores spatial resolution and refines feature representations across multiple scales. The skip connections ensure that high-resolution spatial features from early encoder stages are preserved and effectively fused with deeper semantic representations. This capability is further strengthened by the local depthwise convolutions within the LGL block, which capture fine-grained spatial details and maintain boundary-sensitive information:

11 $$\begin{aligned} \textbf{F}_{local} = \text {DWConv}(\textbf{E}). \end{aligned}$$

In contrast, contextual understanding is facilitated by the transformer-based self-attention mechanism, which models long-range dependencies:

12 $$\begin{aligned} \textbf{F}_{global} = \text {MHSA}(\textbf{Z}), \end{aligned}$$

allowing each spatial location to attend to all other positions in the image.

By integrating these two complementary components, the LGL module effectively bridges local spatial precision and global semantic awareness. This enables the model to produce segmentation outputs that are both boundary-accurate and contextually coherent, leading to robust performance across diverse and challenging medical imaging modalities.

### Model training

Although a wide range of recent architectures including transformer-based (e.g., UNETR^10^, VM-UNet^29^, MA-TransformerV2^30^), automated frameworks (nnU-Net^7^), and advanced hybrid designs were reviewed in Table 1, this study focuses on a subset of representative and practically relevant models, namely SegFormer^12^ , UNet-InceptionV3^1,21^, UNet-MobileNetV2^1,22^, DeepLab-MobileNetV3^23,24^, DeepLab-EfficientNetB0^23,25^ , UNet-EfficientNetB0^1,25^, DeepLab-ResNet101^23,26^, UNet-ResNet101/152^1,26^ and MA-Net^27^. This selection is guided by three considerations. First, these models cover diverse architectural paradigms, including convolutional, encoder–decoder, lightweight, and early local–global hybrid designs, enabling a fair comparison. Second, they employ widely used backbones (e.g., MobileNet^22^, EfficientNet^25^ and ResNet^26^), serving as strong and standardized baselines for evaluating efficiency and generalization. Third, compared to more complex or highly customized methods (e.g., nnU-Net or transformer-heavy models), they offer better reproducibility and controlled complexity. Thus, this curated set ensures a rigorous and practically meaningful benchmark for evaluating the proposed SwiftMSeg framework.

All these models, along with the proposed SwiftMSeg, were trained under a unified experimental setup to ensure a fair and consistent comparison. The goal was to obtain the best-performing version of each model based on training and validation performance, after which the selected models were evaluated on the test datasets. We experimented with Adam and AdamW optimizers with an initial learning rate of 0.001 to assess their impact on performance. A ReduceLROnPlateau scheduler was employed to monitor validation loss and reduce the learning rate by a factor of 0.5 if no improvement was observed for three consecutive epochs. Each model was trained for up to 50 epochs with a fixed random seed of 42 to ensure reproducibility. All models were initialized with ImageNet pre-trained weights and trained on a GPU. Input images were resized to 256*×*256, with a batch size of 2. All images, regardless of modality, were resized to a unified resolution of 256*×*256 to maintain consistent training conditions and enable fair computational comparison among models. Although this resizing strategy may reduce fine-grained spatial details in certain boundary-sensitive tasks, it provides a standardized and computationally efficient evaluation setting. Data loading used 4 workers, with pinned memory enabled, training data shuffled each epoch, and incomplete batches dropped.

To maintain fairness, the preprocessing pipeline, training protocol, and hyperparameters were kept identical across all models. Data augmentation was applied in a controlled manner using horizontal flipping and rotation. More aggressive augmentations were intentionally avoided, as they may distort clinically relevant structures in medical images and lead to unrealistic samples. For the 4-fold cross-validation experiments, the proposed model was trained multiple times using three different random seeds for each fold to account for variability arising from random initialization and stochastic training dynamics. After that, the trained models were tested on an external dataset to evaluate their robustness.

### Evaluation metrics

Real-world clinical deployment requires models that are not only accurate but also computationally efficient and reliable under resource constraints. Therefore, we adopt a comprehensive set of evaluation metrics to jointly assess segmentation performance, diagnostic reliability, computational efficiency, model compactness, and training practicality across diverse medical imaging modalities, including ultrasound, histopathology, colonoscopy and MRI images.

Segmentation performance metrics Segmentation performance was evaluated using conventional pixel-level metrics, including accuracy, precision, recall, F1-score, intersection over union (IoU), and dice coefficient, computed from true positive (TP), true negative (TN), false positive (FP), and false negative (FN) predictions. Higher accuracy indicates better overall pixel-wise classification performance; however, it may be less informative in highly imbalanced segmentation tasks where background pixels dominate. Precision reflects the proportion of correctly predicted foreground pixels, while Recall measures the ability of the model to capture actual foreground regions. The F1-score provides a balanced evaluation of precision and recall, particularly when both false positives and false negatives are important. IoU and Dice coefficient quantify the overlap between predicted and ground truth regions, with dice being relatively more sensitive to small structures and IoU providing a stricter overlap assessment.

*Boundary-based metrics* To assess segmentation performance beyond region overlap, we employ boundary-based metrics that evaluate how accurately the predicted contours align with the ground truth boundaries. These metrics are particularly important in medical image segmentation, where precise boundary delineation is critical for diagnosis and treatment planning. Hausdorff distance (HD) measures the maximum deviation between the predicted and ground truth boundaries:

13 $$\begin{aligned} HD(X, Y) = \max \left\{ \sup _{x \in X} \inf _{y \in Y} d(x,y), \; \sup _{y \in Y} \inf _{x \in X} d(x,y) \right\} \end{aligned}$$

average symmetric surface distance (ASSD) measures the average distance between boundary points:

14 $$\begin{aligned} ASSD(X, Y) = \frac{1}{|X| + |Y|} \left( \sum _{x \in X} \min _{y \in Y} d(x,y) + \sum _{y \in Y} \min _{x \in X} d(x,y) \right) \end{aligned}$$

where *X* and *Y* denote the sets of boundary points in the predicted and ground truth masks, respectively, and *d*(*x*, *y*) represents the Euclidean distance.

In terms of interpretation, lower values of HD and ASSD indicate better boundary alignment. HD is sensitive to outliers and captures the worst-case boundary error, making it useful for detecting extreme segmentation failures. In contrast, ASSD provides a more robust average measure of boundary discrepancy and reflects overall contour agreement.

*Computational efficiency metrics* To evaluate the practicality of deploying segmentation models in real-world clinical environments, we consider computational efficiency metrics. Giga floating-point operations per second (GFLOPs) quantify the total number of floating-point operations required for a single forward pass, reflecting the model’s computational complexity. Lower GFLOPs indicate more computationally efficient models. Latency (ms) measures the time required to process a single input image during inference and indicates the model’s responsiveness. Lower latency is critical for real-time or interactive clinical applications. Throughput (images/s) represents the number of images processed per second and reflects the model’s capability to handle high-volume data, which is essential for large-scale screening.

*Model efficiency metrics* To explicitly measure how efficiently models utilize parameters and memory, we include Accuracy per Million Parameters:

15 $$\begin{aligned} \text {Accuracy per Million Parameters (AMP)} = \frac{\text {Accuracy (\%)}}{\text {Total Parameters (in millions)}} \end{aligned}$$

This metric highlights models that achieve high predictive performance with fewer parameters, favoring lightweight and memory-efficient architectures. Higher values of this metric indicates better trade-offs between performance and resource usage, which is particularly important for deployment on edge devices and resource-constrained clinical systems.

### Reproducibility statement

To ensure reproducibility, all experiments were conducted with a fixed seed, and the same protocol was used for all models. For the 4-fold cross-validation experiment, the proposed model was trained three times with three different seeds, and the results were reported as mean ± standard deviation to capture variability from random initialization and stochastic training. Dataset splits are detailed in Tables 2, with the training, validation and test partitions applied consistently across all experiments. The training protocol, including data preprocessing, augmentation and evaluation procedures, was standardized to enable reliable reproduction of our results.

## Results

### Ablation study

To systematically evaluate the contribution of LGL feature extraction and hierarchical multi-scale decoder in the proposed SwiftMSeg architecture, we conduct an ablation study across four diverse medical imaging datasets, including colonoscopy (Table 3), pathology (Table 4), ultrasound (Table 5), and MRI (Table 6). Four model variants are considered: (A) baseline with standard feature aggregation and decoder, (B) baseline with hierarchical multi-scale (HM) decoder, (C) LGL-based feature aggregation with standard decoder and (D) the complete model integrating both LGL and HM.

Comparing variants A and B across all datasets highlights the importance of the HM decoder. A consistent and significant improvement is observed, e.g., Dice increases from 51.54 to 72.16 on Kvasir-SEG^44^ (Table 3) and from 50.49 to 79.48 on BUS-BRA (Table 5). This demonstrates that multi-scale feature fusion and progressive upsampling are crucial for recovering spatial details and improving segmentation quality. However, despite these gains, performance remains limited without enhanced feature representation, indicating that decoder design alone is insufficient. The impact of the proposed LGL module is evident when comparing variants A and C. Incorporating LGL leads to substantial improvements, particularly on complex datasets. For instance, Dice improves from 51.54 to 90.10 on Kvasir-SEG^44^ and from 50.49 to 90.85 on BUS-BRA^48^. This indicates that the LGL block effectively enhances feature representation by integrating local spatial details with global contextual information. The gains are less noticeable on the pathology dataset (Table 4), suggesting that while LGL improves representation, its effectiveness may depend on dataset characteristics such as texture uniformity and object scale variability.

**Table 3:** Performance of SwiftMSeg backbone with different variants on the colonoscopy Kvasir-SEG^44^ dataset (*LGL* local global local, *HM* hierarchical multi-scale, *Params* parameters, *GFLOPs* *Giga floating-point operations per second*, *Acc * Accuracy). Results are reported as mean ± standard deviation over three runs. The best results are highlighted in Bolditalic.

Type	Feature Aggregation	Decoder	Dice	IoU	Acc	Params(M)	GFLOPs
A	Standard	Standard	51.54 ± 2.49	45.24 ± 1.35	83.66 ± 0.09	***0.485***	***0.085***
B	Standard	HM	72.16 ± 0.36	61.69 ± 0.40	87.60 ± 0.16	1.251	0.472
C	LGL	Standard	90.10 ± 0.52	83.52 ± 1.06	94.79 ± 0.39	3.719	0.554
D	**LGL**	**HM**	***92.16 ± 0.52***	***86.77 ± 1.10***	***95.74 ± 0.46***	**4.485**	**0.940**

**Table 4:** Performance of SwiftMSeg backbone with different variants on the pathology NuInsSeg^46^ dataset (*LGL* local global local, *HM* hierarchical multi-scale, *Params* parameters, *GFLOPs* giga floating-point operations per second, *Acc* Accuracy). Results are reported as mean ± standard deviation over three runs. The best results are highlighted in Bolditalic.

Type	Feature Aggregation	Decoder	Dice	IoU	Acc	Params(M)	GFLOPs
A	Standard	Standard	48.64 ± 0.62	42.99 ± 0.25	81.95 ± 0.08	***0.485***	***0.085***
B	Standard	HM	82.79 ± 1.20	72.57 ± 1.21	90.68 ± 0.15	1.251	0.472
C	LGL	Standard	61.82 ± 2.00	51.91 ± 1.28	84.05 ± 0.11	3.719	0.554
D	**LGL**	**HM**	***89.51 ± 0.93***	***81.27 ± 0.89***	***94.02 ± 0.06***	**4.485**	**0.940**

**Table 5:** Performance of SwiftMSeg backbone with different variants on the ultrasound BUS-BRA^48^ dataset (*LGL* local global local, *HM* hierarchical multi-scale, *Params* Parameters, *GFLOPs* giga floating-point operations per second,* Acc* accuracy). Results are reported as mean ± standard deviation over three runs. The best results are highlighted in Bolditalic.

Type	Feature aggregation	Decoder	Dice	IoU	Acc	Params(M)	GFLOPs
A	Standard	Standard	50.49 ± 0.01	47.35 ± 0.01	91.26 ± 0.01	***0.485***	***0.085***
B	Standard	HM	79.48 ± 0.83	71.74 ± 0.98	94.81 ± 0.28	1.251	0.472
C	LGL	Standard	90.85 ± 0.31	85.19 ± 0.38	97.65 ± 0.04	3.719	0.554
D	**LGL**	**HM**	***92.98 ± 0.11***	***88.35 ± 0.12***	***97.94 ± 0.04***	**4.485**	**0.940**

**Table 6:** Performance of SwiftMSeg backbone with different variants on MRI BRISC^49^ dataset (*LGL* Local global local,* HM* hierarchical multi-scale,* Params* Parameters, *GFLOPs* giga floating-point operations per second, *Acc* Accuracy). Results are reported as mean ± standard deviation over three runs. The best results are highlighted in Bolditalic.

Type	Feature aggregation	Decoder	Dice	IoU	Acc	Params(M)	GFLOPs
A	Standard	Standard	52.20 ± 0.12	50.90 ± 0.17	98.14 ± 0.12	***0.485***	***0.085***
B	Standard	HM	79.83 ± 0.46	73.64 ± 0.54	98.82 ± 0.08	1.251	0.472
C	LGL	Standard	83.67 ± 1.37	77.19 ± 1.39	99.25 ± 0.03	3.719	0.554
D	**LGL**	**HM**	***92.61 ± 0.28***	**88.03 ± 0.24**	***99.57 ± 0.15***	***4.485***	**0.940**

The complete model (variant D) consistently achieved the best performance across all datasets, confirming the complementary roles of LGL and HM. For example, Dice scores reach 92.16 (Kvasir-SEG^44^), 89.51 (NuInsSeg^46^), 92.98 (BUS-BRA^48^), and 92.61 (BRISC^49^), outperforming all other configurations. The improvements over variant C demonstrate that even with strong feature representations, effective multi-scale decoding is necessary for precise boundary reconstruction. Conversely, improvements over variant B indicate that enhanced feature aggregation is critical for capturing discriminative semantic information. While the complete model achieved the highest accuracy, it also introduced a higher computational cost (4.485M parameters and 0.94 GFLOPs). In contrast, the baseline model (A) is extremely lightweight but performs poorly in segmentation. Intermediate configurations (B and C) offer a trade-off between efficiency and accuracy; however, they fail to achieve the optimal balance of the full model. This suggests that integrating LGL and HM is essential to maximize performance while maintaining reasonable computational complexity.

Overall, the ablation results demonstrated that (i) the HM decoder significantly improves spatial reconstruction, (ii) the LGL module enhances feature representation through joint local-global modeling, and iii) their combination yields a complementary effect that leads to robust and accurate segmentation across diverse modalities. These findings validate the design choices of SwiftMSeg and highlight the need to jointly model boundary details and global context for medical image segmentation.

### Segmentation performance analysis

The segmentation performance of the proposed SwiftMSeg model is evaluated using hold-out experiments across four heterogeneous medical imaging datasets: Kvasir-SEG^44^ (colonoscopy), NuInsSeg^46^ (histopathology), BUS-BRA^48^ (ultrasound), and BRISC^49^ (MRI). These datasets represent a wide range of imaging challenges, including illumination variations and specular artifacts in colonoscopy, dense and highly textured cellular structures in histopathology, speckle noise and low contrast in ultrasound, and complex multi-structure anatomical variability in MRI. Despite these substantial differences in image characteristics, SwiftMSeg consistently achieves strong performance across all evaluation metrics, demonstrating robust cross-domain generalization and stable segmentation capability across diverse clinical imaging scenarios.

Table 7 presents the segmentation performance on the colonoscopy dataset, where SwiftMSeg achieved the highest Dice (0.922), IoU (0.867), and accuracy (95.74%), outperforming all competing methods. Compared to the strongest baseline (UNet-Inception, Dice 0.912), the improvement is consistent across overlap-based metrics. More importantly, boundary quality is substantially improved, as reflected by the lowest ASSD (6.875), indicating more precise polyp delineation. This is particularly significant in colonoscopy imaging, where small polyps and specular reflections commonly degrade performance, as observed in weaker results from MA-Net (Dice 0.724) and DeepLab-based variants (dice *łe* 0.910). The SwiftMSeg model also achieves the best overall performance with a Dice score of 0.895 and an IoU of 0.814 on the pathology dataset, as presented in Table 8. While the improvement in Dice compared to UNet-EfficientNetB0 (0.892) and UNet-Inception (0.886) appears marginal, it becomes more evident in boundary-sensitive evaluation. Specifically, SwiftMSeg achieves the lowest Hausdorff Distance (37.184), indicating superior structural alignment in densely packed cellular regions. This reflects improved robustness in distinguishing overlapping nuclei and complex tissue textures, where transformer-only models such as SegFormer exhibit lower Dice (0.885) and reduced stability.

**Table 7:** Performance comparison of segmentation models for colonoscopy dataset (Kvasir-SEG^44^) (TH=throughput, APM=accuracy per million parameters, –=not available). The best results are highlighted in Bolditalic.

Model	Acc. (%)	Dice	IoU	Spec.	HD	ASSD	Params (M)	GFLOPs	Disk (MB)	Latency (ms)	TH	AMP
**Proposed (SwiftMSeg)**	***95.742***	***0.922***	***0.867***	**0.977**	**46.352**	***6.875***	**4.485**	***0.940***	**51.6**	**14.998**	**66.7**	**21.345**
SegFormer	91.047	0.814	0.721	0.975	78.653	15.488	***3.715***	1.692	***42.8***	9.477	105.5	***24.510***
UNet-Inception	95.317	0.912	0.854	***0.984***	46.14	7.502	62.029	16.3	711.2	24.078	41.5	1.536
UNet-MobileNetV2	94.497	0.892	0.827	0.982	47.996	8.826	6.629	3.406	76.3	***8.741***	***114.4***	14.254
DeepLab-MobileNetV3	93.826	0.889	0.818	0.963	52.776	9.644	11.021	2.487	126.5	10.118	98.8	8.513
DeepLab-EfficientNetB0	95.118	0.910	0.850	0.975	***44.401***	7.822	7.305	3.396	81.0	11.528	86.7	13.021
UNet-EfficientNetB0	95.233	0.909	0.850	0.977	47.968	8.015	6.252	2.543	68.9	10.294	97.1	15.233
DeepLab-ResNet101	92.413	0.856	0.774	0.967	61.281	12.290	58.626	60.532	671.9	43.246	23.1	1.576
UNet-ResNet101	92.293	0.853	0.770	0.966	65.549	12.462	51.513	15.592	590.5	13.339	75.0	1.791
UNet-ResNet152	91.744	0.836	0.751	0.966	64.501	14.233	67.157	20.473	769.9	16.542	60.5	1.366
MA-Net	88.346	0.724	0.626	0.981	89.080	37.189	182.076	28.420	2085.1	25.068	39.9	0.485

**Table 8:** Performance comparison of segmentation models for pathology dataset (NuInsSeg^46^) (*TH* throughput, *AMP* accuracy per million parameters, – not available). The best results are highlighted in Bolditalic.

Model	Acc. (%)	Dice	IoU	Spec.	HD	ASSD	Params (M)	GFLOPs	Disk (MB)	Latency (ms)	TH	AMP
**Proposed (SwiftMSeg)**	***94.022***	***0.895***	***0.814***	**0.964**	***37.184***	**2.879**	**4.485**	***0.940***	**51.6**	**15.292**	**65.4**	**20.962**
SegFormer	93.822	0.885	0.805	**0.970**	39.920	2.825	***3.715***	1.692	***42.8***	9.322	107.3	***25.257***
UNet-Inception	93.876	0.886	0.807	0.965	41.321	2.986	62.029	16.3	711.2	23.451	42.6	1.513
UNet-MobileNetV2	93.956	0.889	0.810	0.958	42.648	3.042	6.629	3.406	76.3	***7.909***	***126.4***	14.173
DeepLab-MobileNetV3	89.062	0.805	0.696	0.920	45.297	4.641	11.021	2.487	126.5	9.494	105.3	8.081
DeepLab-EfficientNetB0	91.994	0.860	0.768	0.932	39.837	3.205	7.305	3.396	81.0	11.305	88.5	12.593
UNet-EfficientNetB0	94.011	0.892	***0.814***	0.954	39.249	***2.821***	6.252	2.543	68.9	10.241	97.6	15.037
DeepLab-ResNet101	91.969	0.847	0.753	0.954	47.536	4.974	58.626	60.532	671.9	40.929	24.4	1.568
UNet-ResNet101	93.508	0.879	0.796	0.966	40.880	3.304	51.513	15.592	590.5	12.941	77.3	1.815
UNet-ResNet152	93.726	0.885	0.804	0.957	40.915	3.155	67.157	20.473	769.9	16.617	60.2	1.395
MA-Net	93.990	0.886	0.807	0.968	41.514	3.138	182.076	28.420	2085.1	23.480	42.6	0.516

**Table 9:** Performance comparison of segmentation models for ultrasound dataset (BUS-BRA^48^) (*TH* throughput,* AMP* accuracy per million parameters, – not available).The best results are highlighted in Bolditalic.

Model	Acc. (%)	Dice	IoU	Spec.	HD	ASSD	Params (M)	GFLOPs	Disk (MB)	Latency (ms)	TH	AMP
**Proposed (SwiftMSeg)**	***97.94***	***0.929***	***0.883***	**0.988**	***18.333***	***4.134***	**4.485**	***0.940***	**51.6**	**22.551**	**44.3**	**21.836**
SegFormer	91.972	0.547	0.508	***0.999***	31.786	8.367	***3.715***	1.692	***42.8***	15.928	62.8	***24.759***
UNet-Inception	95.477	0.861	0.796	0.961	48.019	10.554	62.029	16.300	711.2	45.054	22.2	1.539
UNet-MobileNetV2	96.618	0.868	0.804	0.987	66.620	48.888	6.629	3.406	76.3	***12.851***	***77.8***	14.574
DeepLab-MobileNetV3	96.283	0.866	0.795	0.988	34.269	8.591	11.021	2.487	126.5	16.439	60.8	8.736
DeepLab-EfficientNetB0	96.665	0.872	0.808	0.986	19.557	6.325	7.305	3.396	81.0	16.261	61.5	13.233
UNet-EfficientNetB0	96.925	0.885	0.824	0.987	47.727	23.534	6.252	2.543	68.9	18.413	54.3	15.504
DeepLab-ResNet101	95.75	0.822	0.753	0.993	19.865	4.699	58.626	60.532	671.9	45.849	21.8	1.633
UNet-ResNet101	94.531	0.766	0.696	0.977	60.415	36.936	51.513	15.592	590.5	21.958	45.5	1.835
UNet-ResNet152	94.038	0.800	0.718	0.961	52.429	12.778	67.157	20.473	769.9	29.034	34.4	1.400
MA-Net	91.133	0.636	0.567	0.970	60.567	40.120	182.076	28.420	2085.1	39.085	25.6	0.500

**Table 10:** Performance comparison of segmentation models for brain MRI dataset (BRISC^49^) (*TH* throughput, *AMP* accuracy per million parameters, – not available).The best results are highlighted in Bolditalic.

Model	Acc. (%)	Dice	IoU	Spec.	HD	ASSD	Params (M)	GFLOPs	Disk (MB)	Latency (ms)	TH	AMP
**Proposed (SwiftMSeg)**	***99.558***	***0.926***	***0.881***	***0.998***	**12.841**	***2.9919***	**4.485**	***0.940***	**51.6**	**14.468**	**69.1**	**22.196**
SegFormer	99.484	0.905	0.858	**0.998**	14.492	3.7312	***3.715***	1.692	***42.8***	7.779	128.6	***26.781***
UNet-Inception	99.362	0.921	0.876	0.995	15.075	3.3145	62.029	16.300	711.2	22.439	44.6	1.601
UNet-MobileNetV2	99.428	0.912	0.866	0.997	22.305	11.784	6.629	3.406	76.3	***6.702***	***149.2***	14.998
DeepLab-MobileNetV3	99.446	0.901	0.846	***0.998***	***12.604***	3.751	11.021	2.487	126.5	8.102	123.4	9.024
DeepLab-EfficientNetB0	99.338	0.905	0.851	0.996	17.153	4.301	7.305	3.396	81.0	10.054	99.5	13.599
UNet-EfficientNetB0	99.496	0.924	***0.881***	0.997	24.161	14.593	6.252	2.543	68.9	8.939	111.9	15.915
DeepLab-ResNet101	99.324	0.899	0.848	0.996	17.088	4.799	58.626	60.532	671.9	40.439	24.7	1.694
UNet-ResNet101	99.403	0.908	0.862	0.997	14.625	3.6845	51.513	15.592	590.5	11.293	88.6	1.929
UNet-ResNet152	99.353	0.910	0.864	0.995	27.335	13.567	67.157	20.473	769.9	15.056	66.4	1.479
MA-Net	99.040	0.891	0.837	0.993	27.426	10.853	182.076	28.420	2085.1	23.370	42.8	0.544

**Fig. 2:**
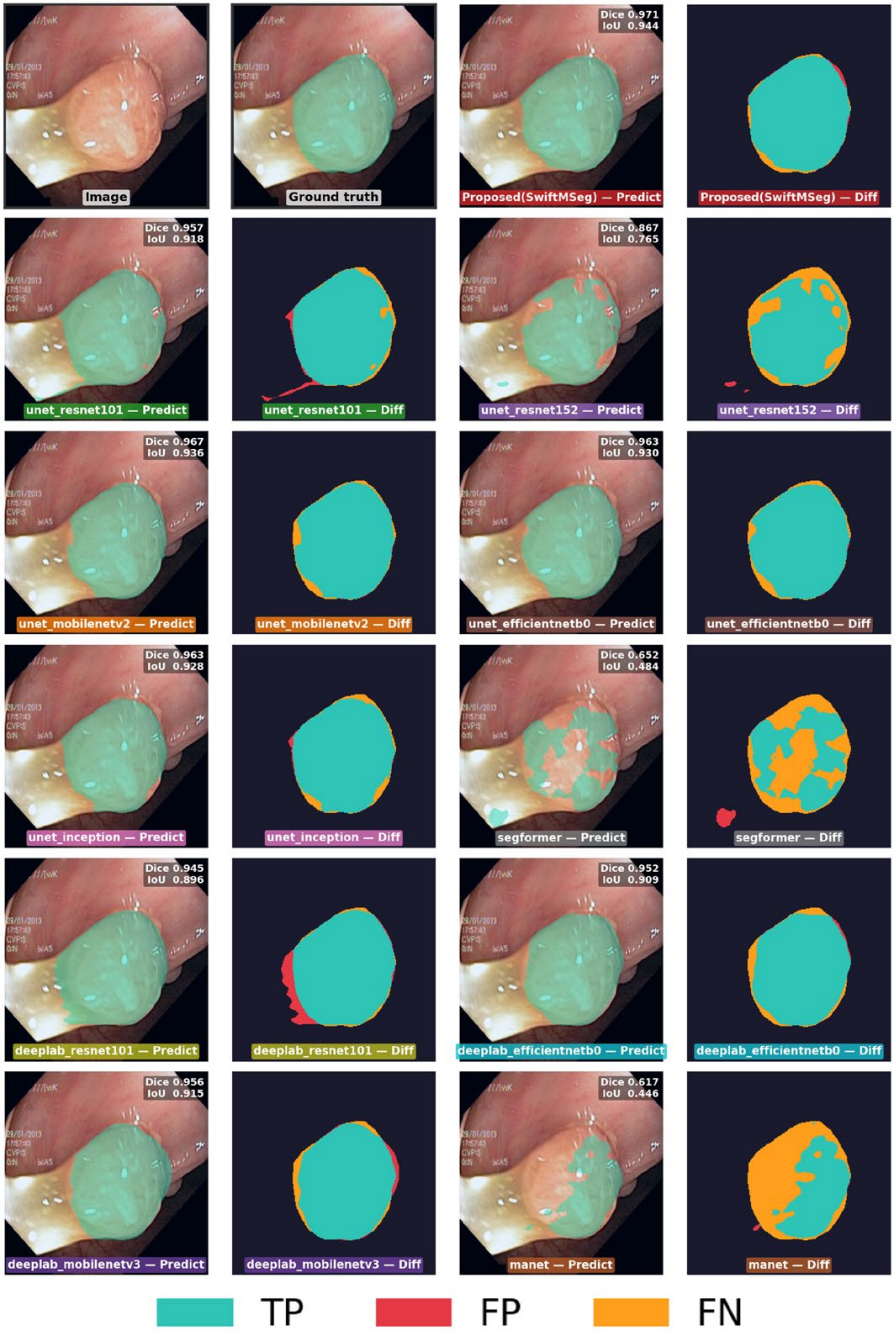
Polyps segmentation in colonoscopy image (Kvasir-SEG^44^) using different models (*TP* true positive, *FP* false positive and *FN* false negative).

**Fig. 3:**
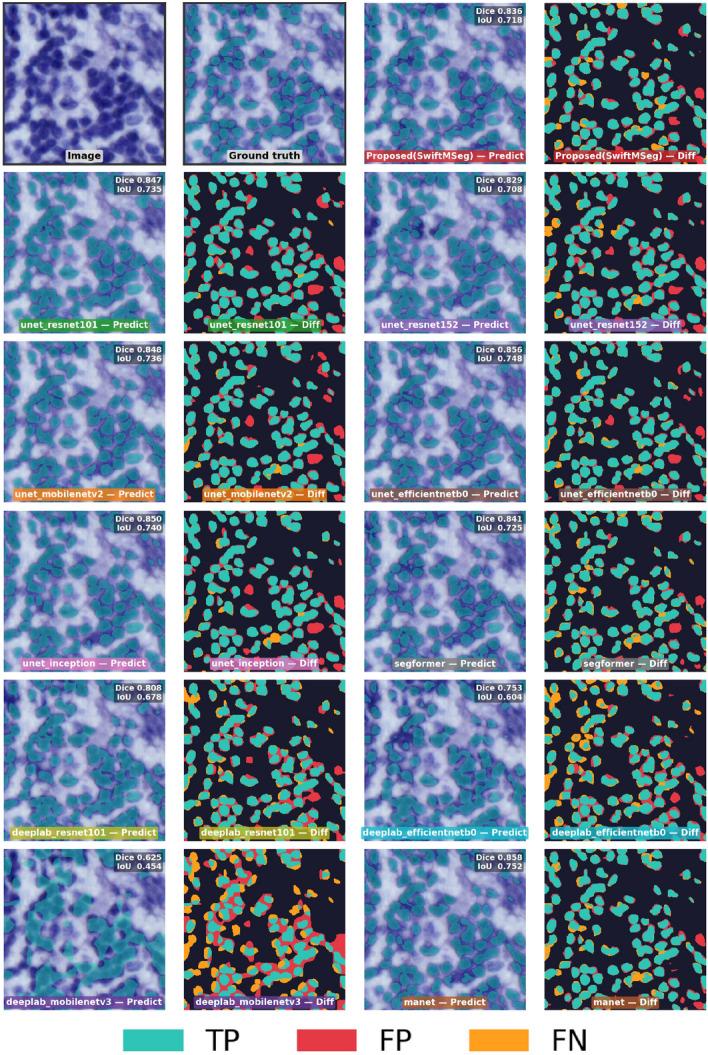
Nuclei segmentation in H&E stained pathology image (NuInsSeg^46^) using different models (*TP* true positive, *FP* false positive and *FN* false negative).

**Fig. 4:**
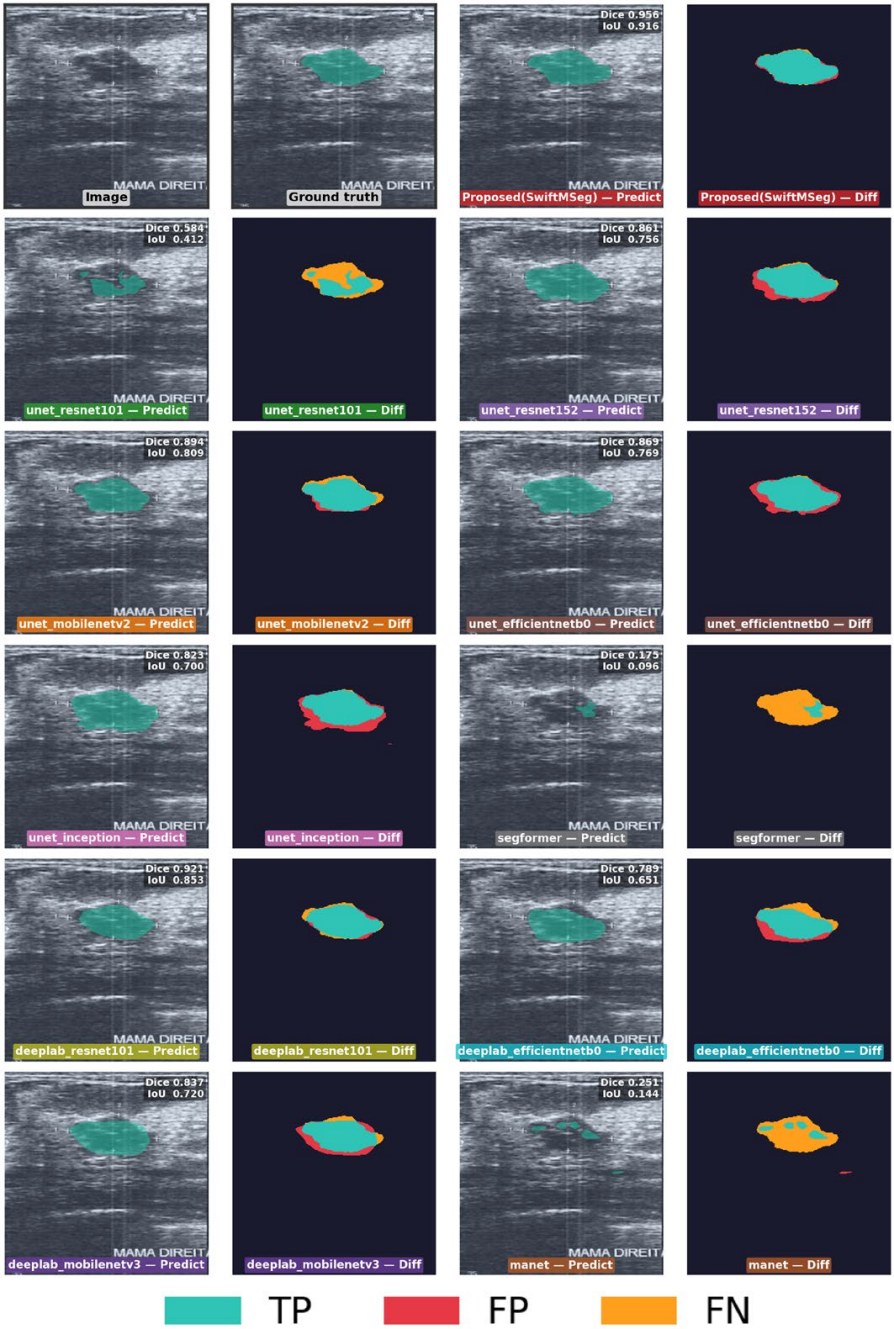
Breast tumor segmentation in ultrasound image (BUS-BRA^48^) using different models (*TP* true positive, *FP* false positive and *FN* false negative).

**Fig. 5:**
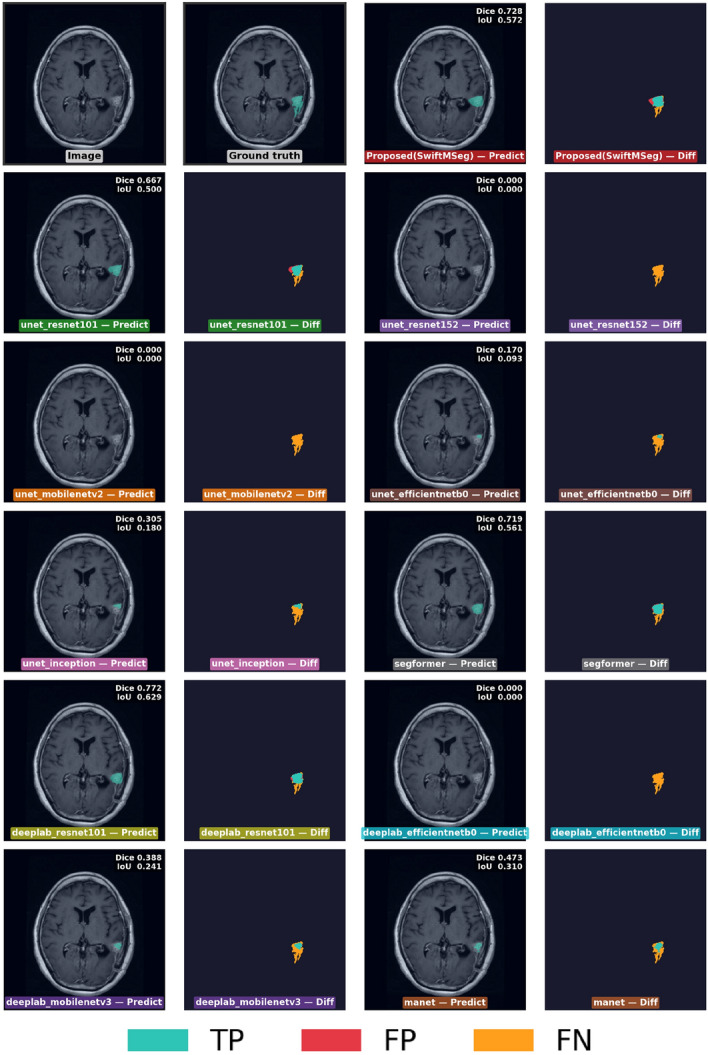
Brain tumor segmentation in MRI image (BRISC^49^) using different models (*TP* true positive, *FP* false positive and *FN* false negative).

The ultrasound images represent the most challenging setting due to speckle noise and low contrast. On the BUS-BRA^48^ dataset, SwiftMSeg demonstrated a clear performance advantage, achieving a Dice score of 0.929 and an IoU of 0.883, outperforming the next best model (UNet-EfficientNetB0, Dice = 0.885), presented in Table 9. The improvement is particularly evident in boundary localization, where ASSD is reduced to 4.134, compared to substantially higher values (exceeding 8–12) observed in most baseline methods. This indicates that SwiftMSeg maintained more accurate lesion boundaries under severe imaging noise and intensity distortions, whereas models such as MANet (Dice 0.636) and UNet-ResNet101 (Dice 0.766) exhibit performance degradation. Table 10 shows the segmentation performance for the MRI images, where structural contrast is comparatively clearer. All models achieve relatively strong performance in MRI images; however, SwiftMSeg still maintains a consistent advantage in both overlap and boundary-based metrics. It achieved a Dice score of 0.926 and an IoU of 0.881, slightly outperforming UNet-EfficientNetB0 (Dice = 0.924). More importantly, it obtains the lowest ASSD (2.9919), indicating highly precise and stable tumor boundary delineation. In contrast, although transformer-based models such as SegFormer achieve competitive accuracy, their lower Dice score (0.905) suggests reduced consistency in region-wise overlap. Overall, these results indicate that SwiftMSeg provided more stable and reliable segmentation performance even in structurally clearer imaging conditions. Fig. 2, 3, 4 and 5 show the segmentation prediction by models for colonoscopy, pathology, ultrasound and MRI images, respectively.

**Fig. 6:**
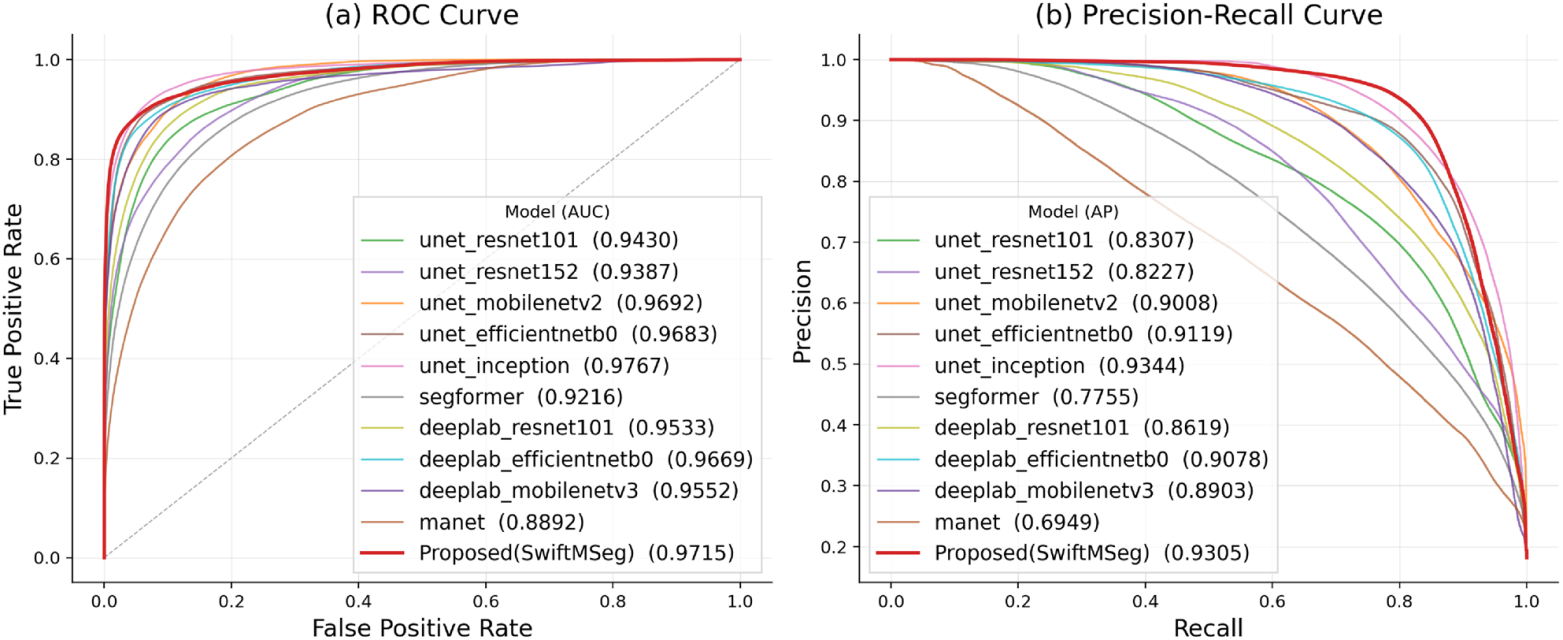
ROC and PR curves of the models for polyps segmentation from colonoscopy images (Kvasir-SEG^44^).

**Fig. 7:**
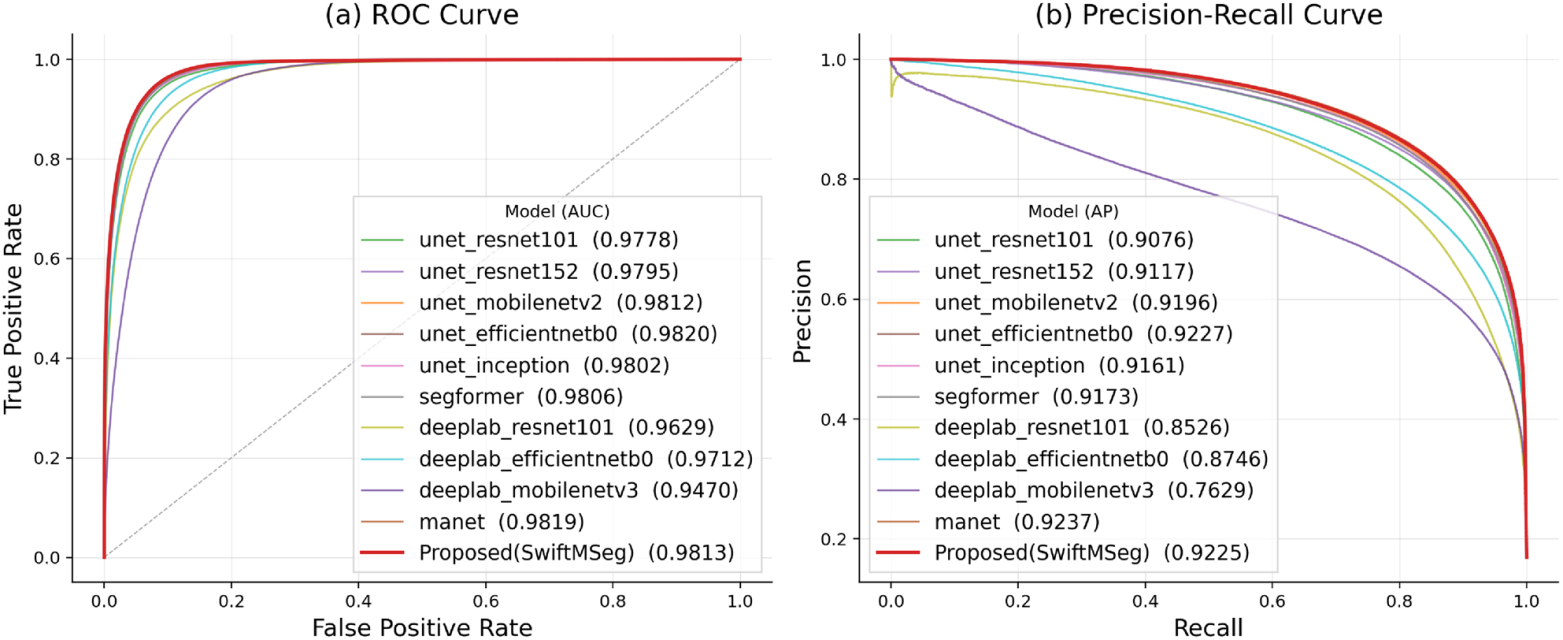
ROC and PR curves of the models for nuclei segmentation from histopathology images (NuInsSeg^46^).

**Fig. 8:**
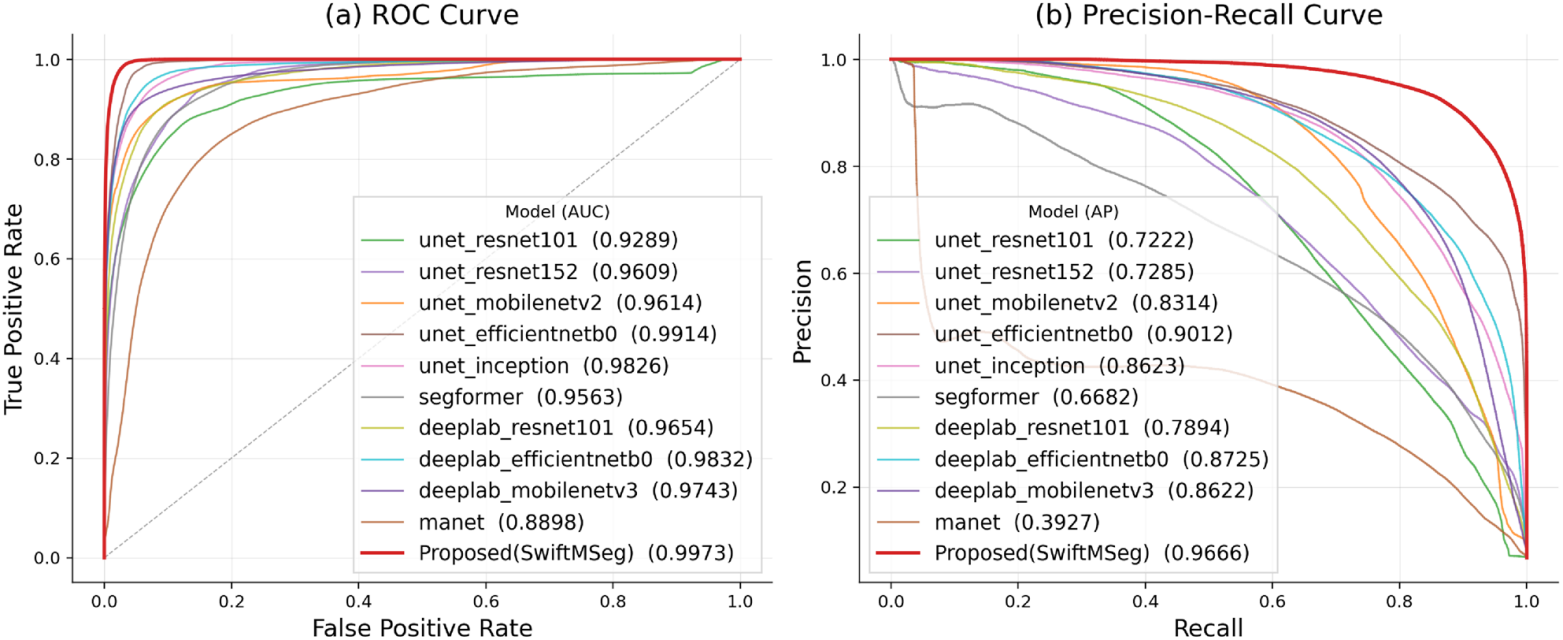
ROC and PR curves of the models for breast tumor segmentation from ultrasound images (BUS-BRA^48^).

**Fig. 9:**
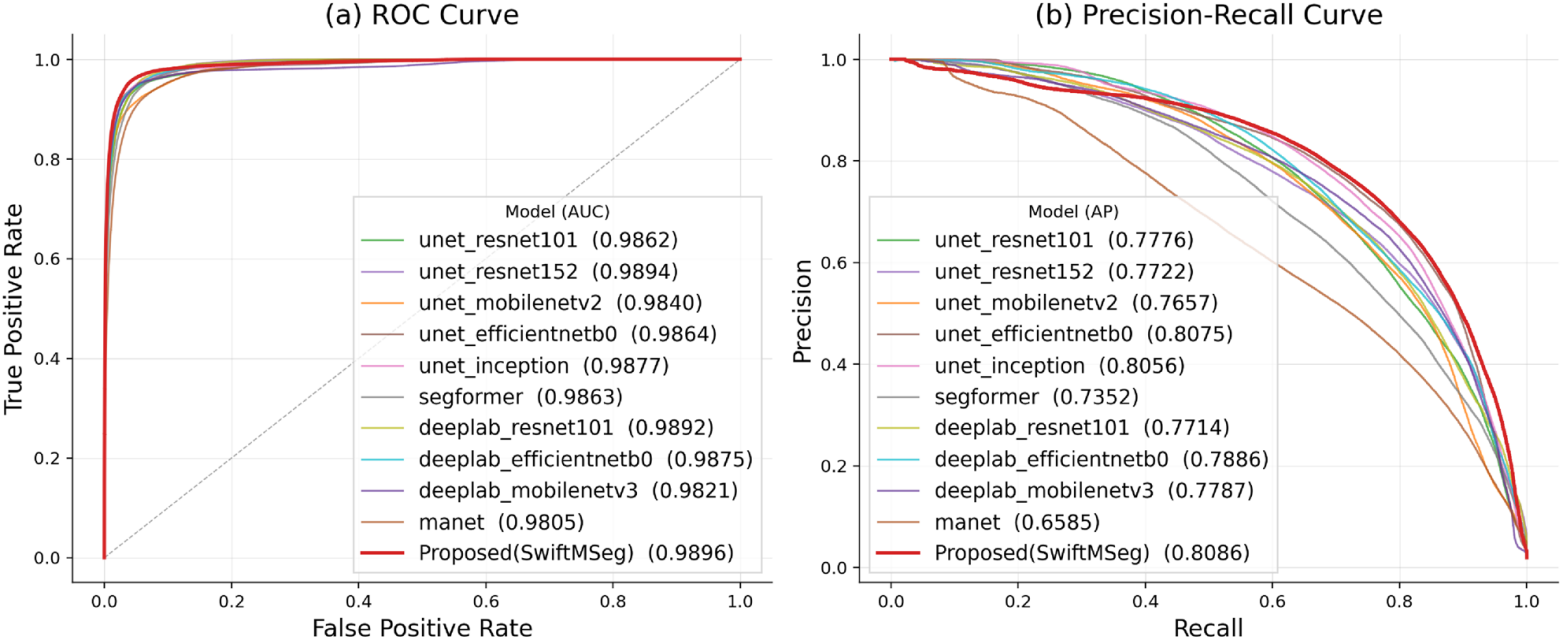
ROC and PR curves of the models for brain tumor segmentation from MRI images (BRISC^49^).

We also plotted the receiver operating characteristics (ROC) curve and precision recall (PR) curve of the models, shown in Fig. 6, 7,8 and 9. They provide a clear insight into the performance of the proposed SwiftMSeg across the four medical imaging modalities. On the Kvasir-SEG^44^ dataset, SwiftMSeg achieves 0.971 (ROC-AUC) and 0.930 (PR-AUC), indicating strong class separation and reliable detection, although slightly below the top-performing model. On NuInsSeg^46^, it attains 0.981 / 0.923, demonstrating nearly identical performance to the best methods. On the more challenging BUS-BRA^48^ ultrasound dataset, SwiftMSeg achieves the highest scores (0.997 / 0.967), reflecting near-perfect separability and highly precise lesion detection with minimal false positives. Similarly, on the Brain Tumor MRI dataset, it leads with 0.990 / 0.809, indicating excellent discrimination and the best precision-recall balance among competing approaches. The AUC values confirm that SwiftMSeg effectively distinguishes lesions from background, while the strong PR-AUC, particularly on ultrasound and MRI, demonstrates its superior reliability in detecting clinically relevant regions under class imbalance.

Across all four datasets, the results consistently indicate three key findings. First, SwiftMSeg consistently achieved either the highest or near-highest Dice and IoU scores across all modalities, demonstrating stable segmentation performance across diverse imaging characteristics. Second, the performance gains are more evident in challenging scenarios, particularly ultrasound and colonoscopy, where noise, low contrast, and imaging artifacts significantly degraded the performance of competing models, highlighting the robustness of the proposed approach under adverse conditions. Third, boundary-sensitive metrics such as ASSD and HD consistently favored SwiftMSeg, indicating more accurate structural alignment and improved lesion localization compared to existing methods. Overall, these observations confirm that SwiftMSeg not only improves overlap-based accuracy but also provides more reliable and geometrically precise segmentation, making it a more robust and clinically dependable solution across heterogeneous medical imaging modalities.

### Computational efficiency analysis

Beyond segmentation accuracy, computational efficiency is a key requirement for clinical deployment, particularly for real-time or resource-constrained medical environments. To evaluate this aspect, we compare models in terms of parameter count $\mathcal {O}(P)$, computational complexity in FLOPs $\mathcal {O}(F)$, memory requirement and inference efficiency, such as latency and throughput. FLOPs can be interpreted as a proxy for computational cost, with higher values generally indicating longer inference time and higher energy consumption. SwiftMSeg is designed to be lightweight, requiring only 4.485M parameters and 0.940 GFLOPs, i.e., $\mathcal {O}(10^6)$ parameter scale. In contrast, conventional CNN-based architectures such as UNet-Inception (62.0M parameters, 16.3 GFLOPs) and UNet-ResNet152 (67.1M parameters, 20.4 GFLOPs) operate at an order of magnitude higher complexity, i.e., $\mathcal {O}(10^7)$ parameter scale. This substantial gap highlights the computational advantage of the proposed method. MANet is the most computationally intensive baseline, with 182M parameters and 28.4 GFLOPs, making it less practical for time-sensitive or edge-deployed medical applications. In terms of memory consumption, SwiftMSeg requires only 51.6 MB of storage, whereas heavy CNN variants such as UNet-Inception (711 MB) and ResNet-based models (approximately 590–770 MB) impose significantly higher storage demands. This difference directly affects deployability in hospital systems, where memory constraints and model portability are critical.

Inference efficiency further reflects the computational advantage of the proposed approach. SwiftMSeg achieves consistently low latency (approximately 7–22 ms depending on modality), whereas larger CNN models such as UNet-Inception require substantially higher inference time (up to *∼*45 ms in ultrasound). Although lightweight transformer-based models such as SegFormer may achieve competitive latency, their inference efficiency is not stable across modalities and is accompanied by significant performance degradation, particularly on challenging datasets such as ultrasound. To better quantify the inference efficiency, we consider the general inference cost formulation:

$$T_{\text {inf}} \propto \mathcal {O}(F) + \mathcal {O}(P),$$

where inference time $T_{\text {inf}}$ increases with both floating-point operations and parameter memory access. Under this formulation, SwiftMSeg minimizes both terms simultaneously, resulting in lower latency and stable throughput across datasets.

From a throughput perspective, SwiftMSeg maintains a balanced performance (approximately 44–69 images/sec), outperforming most computationally heavy architectures. While SegFormer occasionally achieves higher throughput, this comes at the cost of significantly reduced segmentation accuracy (e.g., Dice drop to 0.547 in ultrasound), indicating an unfavorable trade-off between efficiency and clinical reliability. A more comprehensive efficiency indicator is given by the AMP where higher values indicate better utilization of model capacity. Across all datasets, SwiftMSeg consistently achieves high APM values, demonstrating that it delivers strong segmentation performance without relying on excessive model complexity.

Overall, the computational analysis highlights three key findings, which can be clearly observed in the radar charts 10, 11, 12 and 13. First, SwiftMSeg achieved a substantially lower computational complexity compared to classical CNN architectures while maintaining competitive or superior accuracy. Second, it provided a balanced trade-off between latency, throughput and model size, making it suitable for real-time deployment. Third, alternative lightweight models either sacrifice segmentation accuracy or fail to maintain robustness across modalities. Collectively, these results confirm that SwiftMSeg offers a clinically practical and computationally efficient solution for multimodal medical image segmentation.

**Fig. 10:**
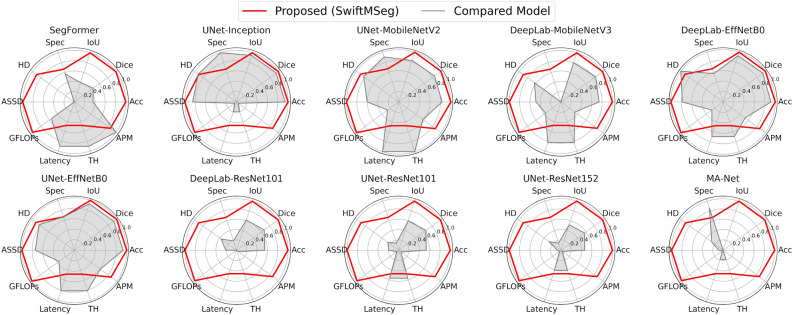
Comparison of the models with the proposed method for colonoscopy dataset (Kvasir-SEG^44^).

**Fig. 11:**
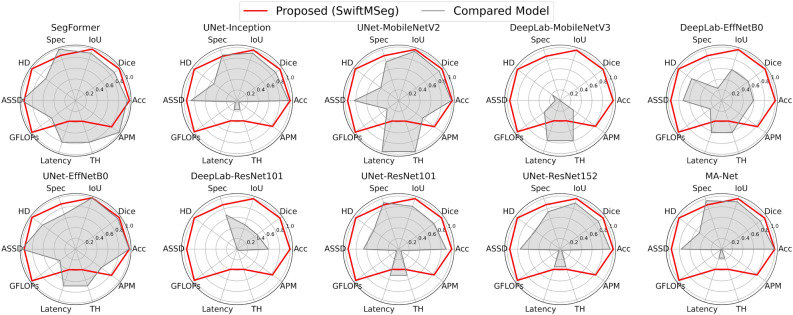
Comparison of the models with the proposed method for pathology dataset (NuInsSeg^46^).

**Fig. 12:**
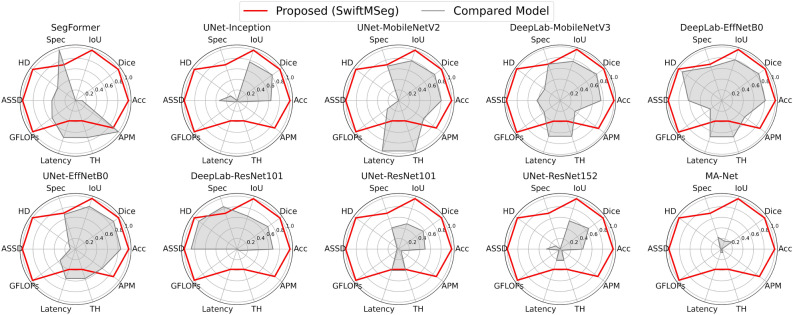
Comparison of the models with the proposed method for ultrasound dataset (BUS-BRA^48^).

**Fig. 13:**
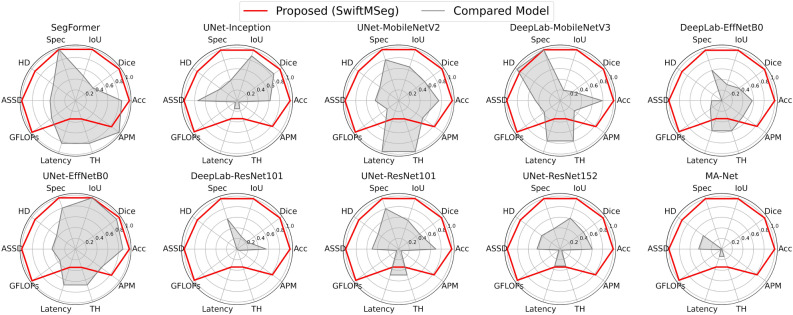
Comparison of the models with the proposed method for MRI dataset (BRISC^49^).

### Robustness analysis

**Fig. 14:**
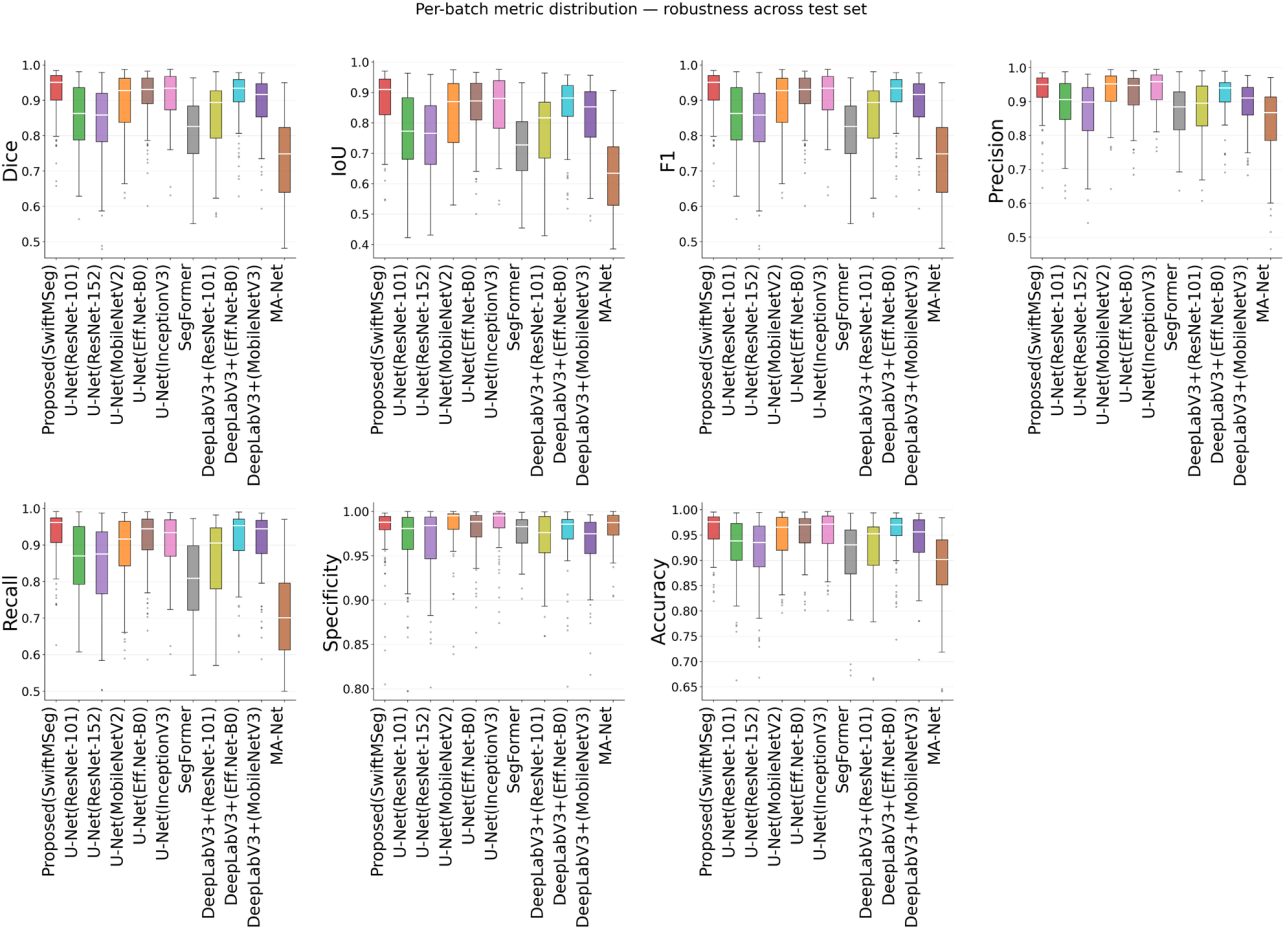
Boxplot distribution for the models for polyps segmentation in colonoscopy (Kvasir-SEG^44^) test images.

**Fig. 15:**
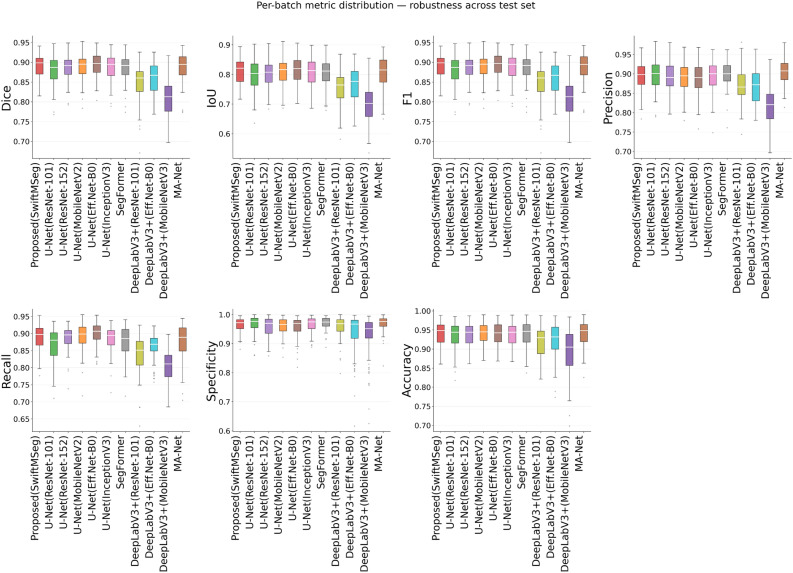
Boxplot distribution for the models for nuclei segmentation in histopathology (NuInsSeg^46^) test images.

**Fig. 16:**
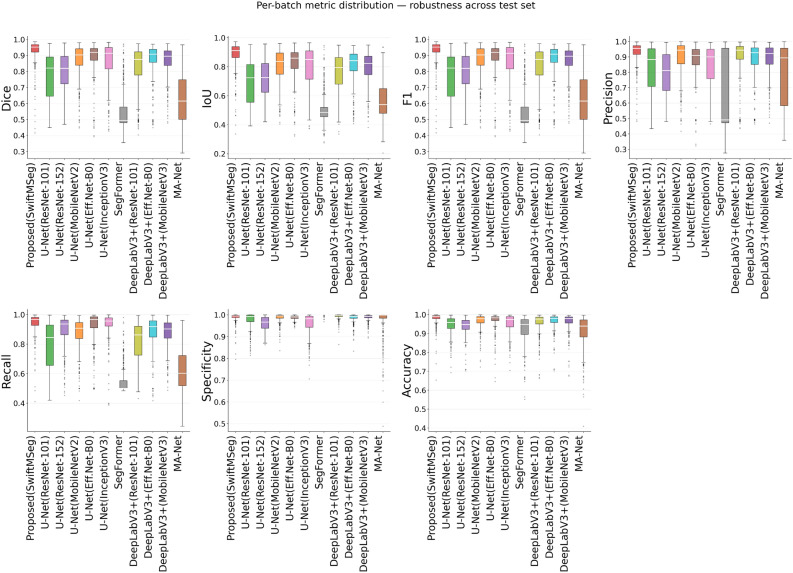
Boxplot distribution for the models for breast tumor segmentation in ultrasound (BUS-BRA^48^) test images.

**Fig. 17:**
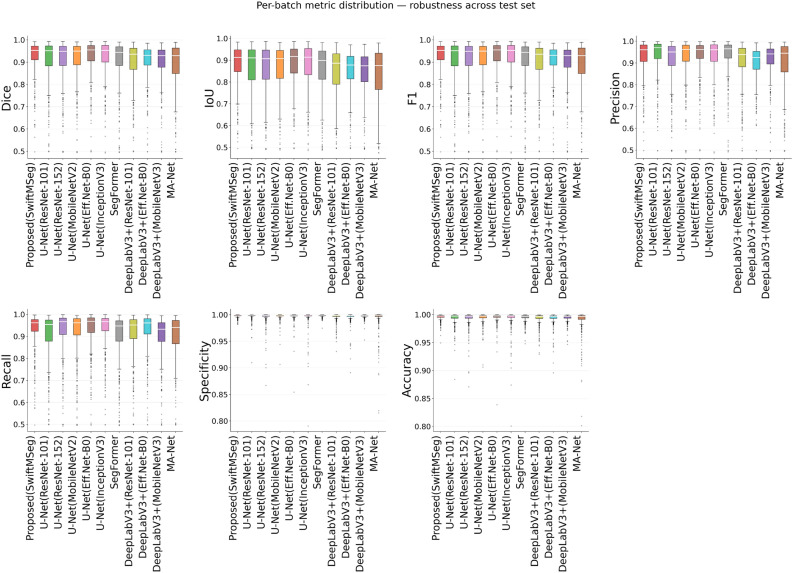
Boxplot distribution for the models for brain tumor segmentation in MRI (BRISC^49^) test images.

To comprehensively evaluate the robustness of the proposed method, experiments were conducted across four dimensions: performance distribution, cross-dataset generalization, cross-validation consistency and robustness to target-scale variation. Firstly, the boxplot distribution provided insights into performance stability, consistency and resistance to failure under diverse imaging conditions. The boxplots in Fig. 14, 15, 16 and 17 demonstrate that the proposed method consistently achieved the highest or near-highest median Dice and IoU across all modalities. More importantly, it shows narrower interquartile ranges (IQRs), particularly in colonoscopy and ultrasound, indicating lower variability compared to models such as SegFormer and UNet-ResNet101, which exhibit wider distributions. The proposed model also maintained higher lower-bound performance, with elevated lower whiskers and fewer extreme outliers in Accuracy and Specificity compared to MANet and DeepLab-MobileNetV3. This indicates stronger resistance to failure cases. In addition, it maintained a balanced Precision–Recall trade-off, achieving high Recall across pathology and ultrasound datasets without a noticeable drop in precision, and remained competitive with UNet-EfficientNetB0. In comparison, MA-Net showed the highest variance and the lowest median, while SegFormer demonstrated performance degradation in ultrasound, with a larger spread and lower central tendency. UNet and DeepLab variants provided relatively stable performance; however, they are consistently outperformed by the proposed method in terms of median scores, IQR, and outlier suppression. Overall, the proposed method demonstrated superior robustness, with higher median performance, reduced variability, and improved resistance to outliers across all modalities.

Overall, the computational analysis highlights three key findings, which can be clearly observed in the radar charts 10, 11, 12 and 13. First, SwiftMSeg achieved a substantially lower computational complexity compared to classical CNN architectures while maintaining competitive or superior accuracy. Second, it provided a balanced trade-off between latency, throughput and model size, making it suitable for real-time deployment. Third, alternative lightweight models either sacrifice segmentation accuracy or fail to maintain robustness across modalities. Collectively, these results confirm that SwiftMSeg offers a clinically practical and computationally efficient solution for multimodal medical image segmentation.

Second, robustness to domain shift was evaluated using four heterogeneous external datasets from different imaging modalities and laboratories, including colonoscopy (CVC-ClinicDB^45^), histopathology (PanNuke^47^), ultrasound (UDIAT^50^), and MRI (SciDB^51^). The external test results are presented in Tables 11, 12, 13 and 14. The proposed method consistently achieved the best or near-best performance across all modalities. For example, it achieved Dice scores of 0.896 (colonoscopy), 0.860 (pathology), 0.850 (ultrasound), and 0.870 (MRI), outperforming most baseline models by clear margins. Importantly, the method also demonstrated strong boundary localization, achieving lower HD values such as 16.43 in MRI and 33.89 in ultrasound, compared to significantly higher values in competing methods (e.g., 25.78 for SegFormer in MRI and 67.63 for UNet-MobileNetV2 in ultrasound). Similarly, ASSD values were consistently lower, indicating more accurate and stable boundary delineation. Although a few models achieved comparable or slightly better performance in isolated metrics (e.g., UNet-EfficientNetB0 in colonoscopy for HD and ASSD), the proposed method maintained the best overall balance across region and boundary-based metrics, demonstrating superior generalization.

**Fig. 18:**
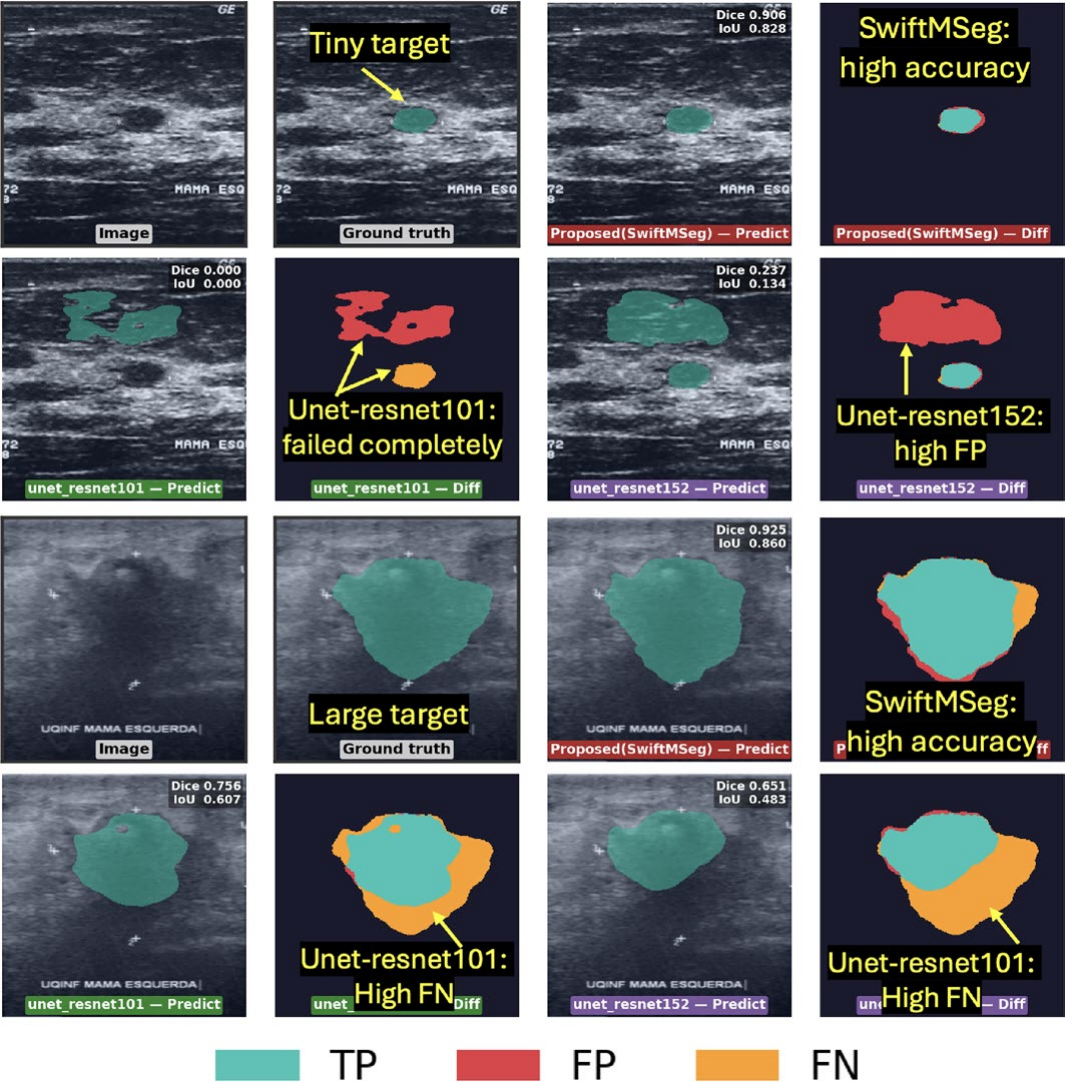
Robustness of the proposed method across the varying target sizes (*TP* true positive, *FP* false positive and *FN* false negative).

Third, a 4-fold cross-validation was performed to assess consistency across different data splits, as presented in Table 15. The proposed method demonstrated highly stable performance across all modalities, as reflected by consistently low standard deviations in Dice and IoU. For instance, Dice variability remained as low as *± 0.0027* in pathology and *± 0.0036* in MRI, while IoU deviations were similarly limited (e.g., *± 0.0032* in pathology and *± 0.0052* in MRI). Even in the more challenging ultrasound modality, the variation remained controlled (Dice *± 0.0133*), indicating robustness under higher data variability. Accuracy further showed near-zero variance in MRI (*± 0.0002*), highlighting strong training stability. These consistently low deviations confirm that the model is not sensitive to data partitioning and can reliably learn robust and generalizable representations across diverse datasets. Finally, robustness to target size variation was analyzed by examining predictions across samples with significantly varying object sizes. The proposed method consistently segmented both small and large targets with high accuracy across all modalities, as shown in Fig. 18. In contrast, baseline models occasionally failed to capture small structures or produced over-segmentation in larger regions. This behavior highlights the effectiveness of the proposed multi-scale design in handling scale variability. Overall, the results demonstrate that the proposed method is robust in terms of performance stability, generalization across datasets, consistency across training splits, and adaptability to scale variations. The consistent improvements across four distinct imaging modalities further confirm its generalization capability and suitability for real-world medical imaging applications. However, larger-scale external validation is still required, particularly since each external test set contained only 80 images.

**Table 11:** Evaluation of the models on an external colonoscopy dataset (CVC-ClinicDB^45^) for polyps segmentation. Results are reported as mean ± standard deviation over three runs. The best results are highlighted in Bolditalic.

Model	Dice	IoU	Accuracy (%)	HD	ASSD
**Proposed (SwiftMSeg)**	***0.896 ± 0.001***	***0.842 ± 0.001***	***96.770 ± 0.000***	***47.259***	***9.980***
unet_efficientnetb0	0.861 ± 0.001	0.795 ± 0.001	**96.840 ± 0.400**	**33.176**	**7.140**
unet_mobilenetv2	0.858 ± 0.004	0.791 ± 0.002	96.800 ± 0.500	53.011	14.801
unet_inception	0.858 ± 0.005	0.789 ± 0.010	95.030 ± 0.700	56.161	15.898
deeplab_mobilenetv3	0.852 ± 0.003	0.778 ± 0.004	95.650 ± 0.700	57.180	14.718
deeplab_efficientnetb0	0.850 ± 0.005	0.786 ± 0.005	96.210 ± 0.000	38.209	9.189
segformer	0.798 ± 0.004	0.706 ± 0.004	94.580 ± 0.100	78.465	18.277
deeplab_resnet101	0.793 ± 0.002	0.710 ± 0.002	94.230 ± 0.000	60.790	15.940
unet_resnet152	0.770 ± 0.005	0.691 ± 0.003	94.480 ± 0.000	69.597	16.848
unet_resnet101	0.737 ± 0.001	0.662 ± 0.001	93.880 ± 0.000	72.518	20.261
manet	0.660 ± 0.003	0.584 ± 0.003	92.640 ± 0.600	74.692	28.105

**Table 12:** Evaluation of the models on an external histopathology dataset (PanNuke^47^) for tumor segmentation. Results are reported as mean ± standard deviation over three runs. The best results are highlighted in Bolditalic.

Model	Dice	IoU	Accuracy (%)	HD	ASSD
**Proposed (SwiftMSeg)**	***0.860 ± 0.004***	***0.783 ± 0.003***	***93.831 ± 0.100***	***37.9767***	***4.6069***
unet_resnet152	0.844 ± 0.009	0.770 ± 0.008	93.467 ± 0.000	40.012	4.912
segformer	0.824 ± 0.006	0.742 ± 0.005	92.686 ± 0.200	50.957	6.816
unet_mobilenetv2	0.823 ± 0.003	0.742 ± 0.003	92.399 ± 0.200	54.267	6.846
unet_efficientnetb0	0.818 ± 0.003	0.745 ± 0.003	92.787 ± 0.300	54.342	9.423
unet_resnet101	0.815 ± 0.004	0.732 ± 0.003	92.236 ± 0.000	49.256	5.925
unet_inception	0.810 ± 0.002	0.730 ± 0.001	92.325 ± 0.100	67.636	11.825
manet	0.804 ± 0.007	0.725 ± 0.009	92.227 ± 0.300	53.868	7.640
deeplab_efficientnetb0	0.789 ± 0.009	0.706 ± 0.006	90.583 ± 0.000	62.624	9.843
deeplab_mobilenetv3	0.763 ± 0.001	0.663 ± 0.001	88.977 ± 0.000	55.809	9.590
deeplab_resnet101	0.681 ± 0.002	0.573 ± 0.004	75.375 ± 0.300	60.355	8.110

**Table 13:** Evaluation of the models on an external ultrasound dataset (UDIAT^50^) for tumor segmentation. Results are reported as mean ± standard deviation over three runs. The best results are highlighted in Bolditalic.

Model	Dice	IoU	Accuracy (%)	HD	ASSD
**Proposed (SwiftMSeg)**	***0.850 ± 0.005***	***0.794 ± 0.005***	***97.549 ± 0.000***	***33.889***	***9.879***
unet_efficientnetb0	0.840 ± 0.005	0.768 ± 0.005	97.189 ± 0.000	44.293	10.784
deeplab_resnet101	0.820 ± 0.007	0.742 ± 0.006	97.092 ± 0.000	43.182	13.054
deeplab_efficientnetb0	0.814 ± 0.005	0.736 ± 0.002	97.052 ± 0.000	40.964	13.922
unet_mobilenetv2	0.797 ± 0.001	0.715 ± 0.002	95.668 ± 0.000	67.627	14.703
deeplab_mobilenetv3	0.781 ± 0.001	0.702 ± 0.003	96.223 ± 0.000	60.395	16.923
unet_inception	0.760 ± 0.003	0.677 ± 0.004	93.439 ± 0.000	75.905	17.633
unet_resnet101	0.744 ± 0.003	0.657 ± 0.002	93.793 ± 0.000	74.571	18.005
unet_resnet152	0.708 ± 0.001	0.617 ± 0.003	91.185 ± 0.000	80.329	19.799
manet	0.688 ± 0.005	0.609 ± 0.006	91.330 ± 0.000	97.539	28.847
segformer	0.663 ± 0.010	0.604 ± 0.008	96.276 ± 0.000	180.364	166.725

**Table 14:** Evaluation of the models on an external MRI dataset (SciDB^51^) for brain tumor segmentation. Results are reported as mean ± standard deviation over three runs. The best results are highlighted in Bolditalic.

Model	Dice	IoU	Accuracy (%)	HD	ASSD
**Proposed (SwiftMSeg)**	***0.870 ± 0.004***	***0.810 ± 0.003***	***99.507 ± 0.000***	***16.426***	***4.221***
segformer	0.801 ± 0.005	0.738 ± 0.008	99.315 ± 0.000	25.783	10.090
deeplab_mobilenetv3	0.767 ± 0.004	0.702 ± 0.002	99.359 ± 0.200	17.451	7.558
deeplab_efficientnetb0	0.765 ± 0.007	0.704 ± 0.003	98.959 ± 0.400	33.235	11.358
unet_inception	0.743 ± 0.004	0.684 ± 0.008	98.335 ± 0.600	78.770	49.579
unet_efficientnetb0	0.722 ± 0.012	0.670 ± 0.007	99.258 ± 0.000	174.353	162.436
unet_mobilenetv2	0.703 ± 0.007	0.654 ± 0.007	98.821 ± 0.000	107.305	95.084
manet	0.649 ± 0.009	0.601 ± 0.010	98.794 ± 0.200	39.501	15.360
unet_resnet101	0.647 ± 0.004	0.607 ± 0.006	99.180 ± 0.200	193.496	186.445
unet_resnet152	0.622 ± 0.003	0.586 ± 0.004	98.567 ± 0.000	144.762	127.98
deeplab_resnet101	0.598 ± 0.012	0.563 ± 0.007	97.633 ± 0.000	121.404	97.522

**Table 15:** 4-fold cross validation results of the proposed model (SwiftMSeg) on four different medical image datasets.

Metric	Colonoscopy	Pathology	Ultrasound	MRI
Accuracy	*0.9537 ± 0.0021*	*0.9470 ± 0.0030*	*0.9592 ± 0.0029*	*0.9957 ± 0.0002*
Dice	*0.9086 ± 0.0064*	*0.8895 ± 0.0027*	*0.8502 ± 0.0133*	*0.9224 ± 0.0036*
IoU	*0.8481 ± 0.0090*	*0.8125 ± 0.0032*	*0.7720 ± 0.0158*	*0.8745 ± 0.0052*

### Statistical significance

We conducted statistical analysis based on per-image segmentation scores (Dice, IoU, and accuracy) across external test datasets to assess the statistical significance of performance improvements. Since each model was evaluated on the same set of images, we used paired statistical tests to ensure a fair, sample-wise comparison. We applied paired *t*-tests to assess whether the mean difference between the two methods is statistically significant under the assumption of normality. The test statistic is computed as:

$$t = \frac{\textrm{mean}(d)}{\textrm{std}(d) / \sqrt{n}}$$

where *d* represents the per-image differences and *n* is the number of samples.

We also used the Wilcoxon signed-rank test, a non-parametric alternative that does not assume normality and is more robust for medical imaging data. In addition, Cohen’s *d* was used to quantify the effect size:

$$d = \frac{\mu _1 - \mu _2}{\sigma }$$

where $\mu _1$ and $\mu _2$ are the mean performances and *σ* is the pooled standard deviation. This combination ensures that improvements are statistically valid and practically meaningful.

On the colonoscopy external test set, SwiftMSeg achieved higher performance (Dice: 0.896, IoU: 0.843, Accuracy: 0.967) compared to all baselines. The paired *t*-test produced p-values ranging from $1.77 \times 10^{-26}$ to $1.76 \times 10^{-11}$, while Wilcoxon p-values ranged from $1.19 \times 10^{-14}$ to $1.76 \times 10^{-11}$, confirming strong statistical significance. Effect size analysis showed large to very large improvements, particularly against MA-Net (Dice *d = 1.79*, IoU *d = 1.90*) and SegFormer (*d ≈ 0.87*–0.99). Even for strong baselines such as DeepLab-ResNet101, effect sizes remained high (*d ≈ 0.81*–0.89). A few lightweight models showed smaller effect sizes (*d ≈ 0.35*–0.43), but improvements remained statistically significant. On the pathology dataset, SwiftMSeg outperformed all models (Dice: 0.851, IoU: 0.778, Accuracy: 0.938). The paired *t*-test yielded p-values ranging from $2.24 \times 10^{-34}$ to $4.62 \times 10^{-3}$, while Wilcoxon p-values ranged from $9.13 \times 10^{-15}$ to $9.12 \times 10^{-1}$. Very large effect sizes were observed for DeepLab-MobileNetV3 (Dice *d = 1.91*, IoU *d = 2.37*, accuracy *d = 1.04*). Strong gains were also observed for DeepLab-EfficientNetB0 and DeepLab-ResNet101 (*d ≈ 0.94*–1.20). SegFormer and UNet-Inception showed large improvements, particularly for IoU (*d > 0.9*), while moderate gains were observed for UNet-based models (*d ≈ 0.6*–0.8). UNet-ResNet152 showed smaller effect sizes (*d < 0.3*) and higher p-values, indicating marginal differences.

On the UDIAT ultrasound dataset, SwiftMSeg achieved improved performance (Dice: 0.846, IoU: 0.794, accuracy: 0.975). The paired *t*-test p-values ranged from $1.00 \times 10^{-18}$ to $1.80 \times 10^{-5}$, and Wilcoxon p-values ranged from $1.00 \times 10^{-12}$ to $2.00 \times 10^{-5}$. Effect size analysis revealed large improvements for most models, including UNet-ResNet152 (IoU *d = 1.28*, Accuracy *d = 1.44*) and SegFormer (*d ≈ 1.14*–1.20). Moderate gains were observed for UNet-MobileNetV2 and DeepLab variants (*d ≈ 0.5*–0.8), while smaller effect sizes (*d < 0.3*) were observed in a few cases, though results remained statistically significant. On the MRI external dataset, the proposed method achieved superior results (Dice: 0.858, IoU: 0.808, accuracy: 0.995). The paired *t*-test produced p-values ranging from $7.77 \times 10^{-21}$ to $1.81 \times 10^{-4}$, while Wilcoxon p-values ranged from $5.87 \times 10^{-14}$ to $6.46 \times 10^{-5}$. Large to very large effect sizes were observed for DeepLab-ResNet101 (Dice *d = 1.40*, IoU *d = 1.34*) and MA-Net (*d ≈ 1.41*–1.42). UNet-ResNet variants also showed strong improvements (*d ≈ 1.19*–1.27), while moderate gains were observed for SegFormer and UNet-Inception (*d ≈ 0.5*–0.7). Smaller but significant gains were observed in some accuracy comparisons (*d < 0.4*).

Across all modalities, the proposed SwiftMSeg model consistently achieved statistically significant improvements (typically *p < 0.001*), supported by large effect sizes (*d > 0.8* in most cases). The agreement between the paired *t*-test and the Wilcoxon test confirms robustness to distributional assumptions, demonstrating that the improvements are statistically significant, practically meaningful, and generalizable across diverse medical imaging domains.

### Failure and critical cases

**Fig. 19:**
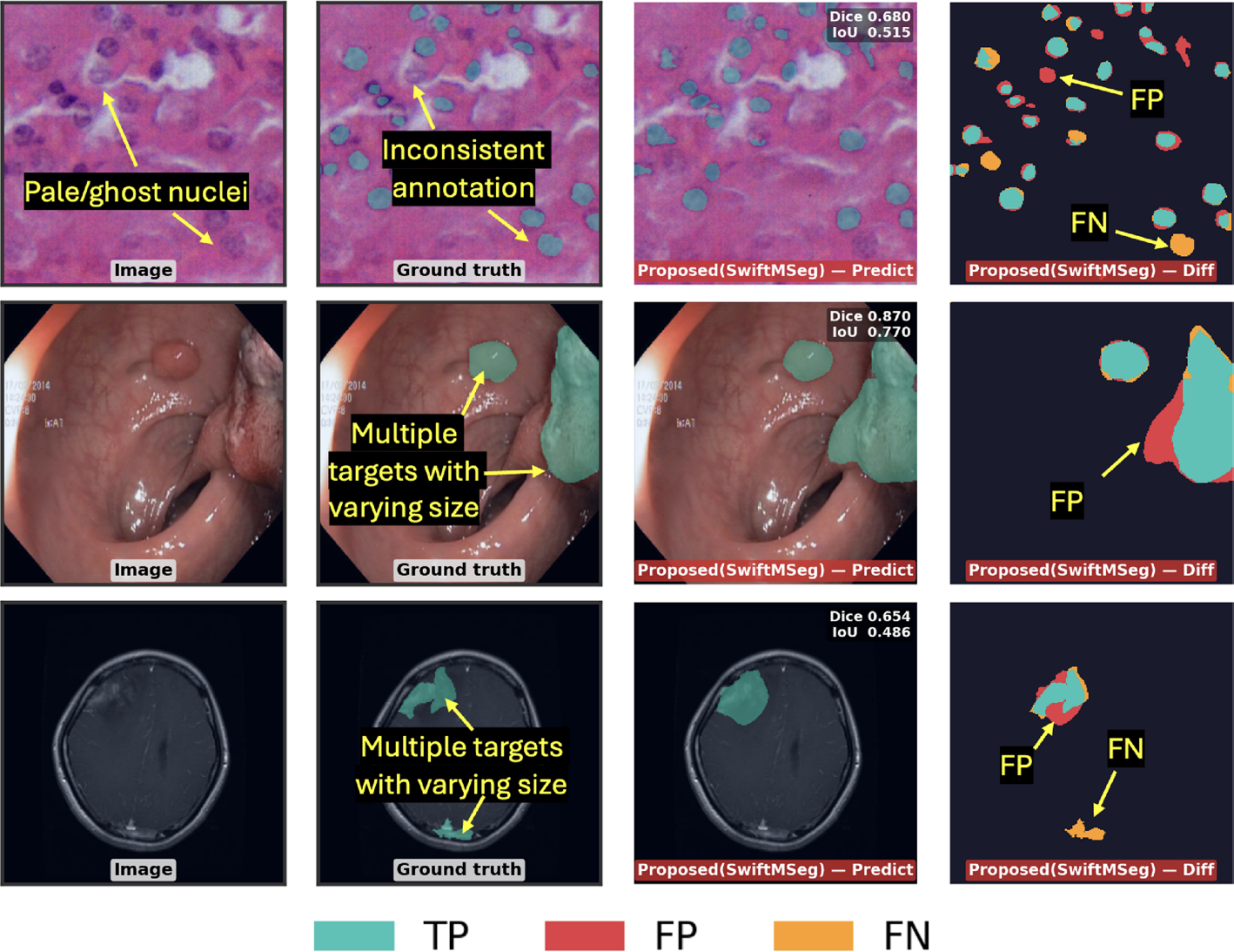
Scenarios where the segmentation models fails to achieve sufficiently accuracy (*TP* true positive, *FP* false positive and *FN* false negative).

Despite the overall strong performance, the proposed method, along with other models, exhibits limitations under certain challenging conditions. By scrutinizing the model predictions, two patterns were identified in which performance drops from its average behavior, as illustrated in Fig. 19. First, in colonoscopy images with multiple polyps of varying sizes, the model performance degrades slightly. The presence of numerous structures with varying scales increases visual complexity, leading to confusion between true polyp regions and background tissues. As a result, the model tends to over-segment, leading to a substantial increase in false-positive pixels compared to its average performance. Second, the models fail to reliably detect nuclei with low contrast or pale staining. In the presence of such ghost nuclei, both false positive and false negative rates increase considerably. This issue is further aggravated by inconsistent annotations, where visually similar nuclei are selectively labeled. Such label noise introduces ambiguity during training, causing unstable predictions and reduced generalization. These limitations highlight the need for improved robustness to visual variability and more consistent annotation strategies.

## Discussion

This study addresses the challenge of simultaneously achieving fine boundary localization and robust contextual understanding in lightweight medical image segmentation models. The experimental results of our study suggest that this trade-off can be resolved by integrating feature representation and decoding strategies. The proposed SwiftMSeg framework demonstrated that combining local–global feature aggregation with hierarchical multi-scale refinement provides a practical solution to this problem. Importantly, this design reflects a shift from treating boundary refinement and contextual modeling as separate objectives to a unified framework that co-optimizes both, enabling more reliable segmentation across diverse imaging conditions. This is evidenced by the consistently high Dice scores of the proposed method, achieved across modalities (0.896 in colonoscopy, 0.860 in pathology, 0.850 in ultrasound, and 0.870 in MRI on external datasets), along with improved boundary localization reflected by lower HD (e.g., 16.43 in MRI) and reduced ASSD. Furthermore, these improvements were found to be statistically significant (*p < 0.001*) with large effect sizes across modalities, confirming that the gains are both robust and practically meaningful.

The ablation analysis offers important insight into this design. The hierarchical decoder improves spatial detail recovery, while the LGL module enhances contextual reasoning by modeling long-range dependencies. However, neither component alone is sufficient to achieve optimal performance. The consistent improvement of the complete model across all datasets suggests that boundary precision and contextual understanding are interdependent and their joint optimization is critical for robust segmentation. This observation further indicates that improvements in feature representation must be effectively translated through appropriate decoding mechanisms, highlighting the importance of architectural synergy rather than isolated module design. This is supported by the ablation results, where the hierarchical decoder alone improves Dice from 51.54 to 72.16 on Kvasir and from 50.49 to 79.48 on BUS-BRA, while the LGL module increases Dice to 90.10 and 90.85, respectively; the complete model further achieves the highest performance, reaching 92.16, 89.51, 92.98, and 92.61 across colonoscopy, pathology, ultrasound, and MRI datasets, confirming the complementary benefits of both components.

A key strength of SwiftMSeg is its consistent performance across diverse imaging modalities, including colonoscopy, pathology, ultrasound, and MRI. These modalities present fundamentally different challenges, such as noise, texture complexity, and structural variability. The ability of the model to maintain high Dice scores alongside improved boundary metrics (e.g., lower HD and ASSD) indicates that it effectively captures both global structure and fine-grained details. This suggests that the proposed framework learns modality-agnostic representations that generalize across varying imaging characteristics. At the same time, the relatively modest gains in pathology suggest that the benefits of global context modeling may vary with image characteristics, particularly in highly repetitive or texture-dominated data, where local texture cues may outweigh long-range dependencies.

The external validation further highlights the potential cross-domain robustness of the proposed approach. Unlike most models that exhibit performance degradation under domain shift, SwiftMSeg maintained consistent improvements across datasets collected from different laboratories and imaging modalities. The statistical analysis further supports this observation, indicating that the improvements are not only consistent but also statistically significant with large effect sizes. This consistency across both parametric and non-parametric statistical tests strengthens the reliability of the findings and suggests that the model is capable of learning transferable representations beyond dataset-specific characteristics. This aspect is particularly important in medical imaging applications, where robustness across varying data distributions is essential. Nevertheless, it should be noted that each external validation subset contained a relatively limited number of samples (80 images per modality). Therefore, while the results demonstrate promising cross-domain performance, larger-scale multi-center evaluations will be necessary to establish stronger evidence for broader clinical generalizability.

From a practical perspective, the results emphasize that high segmentation performance does not necessarily require high computational complexity. SwiftMSeg achieved a substantial reduction in parameters and FLOPs while maintaining or surpassing the accuracy of heavier CNN-based models. In contrast to some lightweight or transformer-based approaches that compromise robustness for efficiency, SwiftMSeg demonstrates that a well-balanced architectural design can achieve both. This highlights the importance of considering deployment constraints alongside accuracy during model development.

Despite these strengths, the analysis of failure cases reveals persistent challenges. The model shows limitations in scenarios involving multiple objects with varying scales and in low-contrast regions with ambiguous annotations, leading to over-segmentation or missed detections. These issues suggest that, beyond architectural improvements, factors such as annotation quality and data variability play a significant role in segmentation performance in medical imaging. Another limitation of this study is the use of a fixed input resolution (256*×*256) across all imaging modalities. While this facilitates consistent evaluation and computational efficiency, downsampling may reduce fine structural information, particularly in pathology and boundary-sensitive segmentation tasks. Future work may investigate adaptive-resolution or patch-based strategies to better preserve high-resolution spatial details.

In summary, the findings clearly demonstrate the underlying premise of this study that effective medical image segmentation in real-world settings requires a balanced integration of boundary refinement, contextual modeling, and computational efficiency. SwiftMSeg demonstrates that such a balance is achievable, while also highlighting remaining challenges related to domain variability and visual ambiguity that require further investigation.

## Conclusions

This paper presented SwiftMSeg, a lightweight segmentation framework that effectively addresses the boundary–context trade-off through local–global feature aggregation and hierarchical multi-scale decoding. The model demonstrates robust, accurate, and domain-independent performance across diverse modalities while maintaining high computational efficiency. These results highlight its suitability for real-world clinical applications. Future work will focus on improving robustness in challenging scenarios and incorporating explainable AI techniques to enhance trust and transparency for clinical decision support.

## Data Availability

The datasets used in this research is publicly available and can be accessed via these links: Kvasir-SEG, CVC-ClinicDB, NuInsSeg, PanNuke, BUS-BRA, UDIAT, BRISC and SciDB. The code used in this study is publicly available at: GitHub Code
